# Supplementary material for: Human Filariasis in Africa (2000–2025): Changing epidemiology, uneven diagnostic progress, and persistent neglect
Source: PLoS Negl Trop Dis. 2026 Apr 7;20(4):e0014200. doi: 10.1371/journal.pntd.0014200 (PMC13082706; doi:10.1371/journal.pntd.0014200)
Supplement: S1 Appendix — (DOCX) [file pntd.0014200.s001.docx]

# **S1 Appendix**

## **S1.1. Search Strategy**

Search syntax was adapted for each database according to available Boolean operators and field tags. Animal studies were excluded when possible using database filters. Where no language filters were available (e.g., African Index Medicus), records in English were selected manually. Although the search strategy included terms related to Dirofilaria spp. and dirofilariasis, no studies met the inclusion criteria for this pathogen in the African context. As a result, no prevalence data on human dirofilariasis were included in the final analysis.

**Table A**  Search strategy (categories connected by ‘AND’).

| **Epidemiological  Parameters** | **Disease** | **Country** |
| --- | --- | --- |
| (prevalence OR incidence OR epidemiology OR occurrence OR frequency) | filariasis OR („lymphatic filariasis” OR elephantiasis OR „*Wuchereria bancrofti*” OR „*W. bancrofti*” OR „*Brugia malayi*” OR „*B. malayi*”) OR OR (onchocerciasis OR "river blindness" OR "*Onchocerca volvulus*" OR „*O. volvulus*”) OR (loiasis OR "african eye worm" OR "*Loa loa*" OR „*L. loa*”) OR (mansonellosis OR "*Mansonella* spp.") OR (dirocilariasis OR "*Dirofilaria* spp.") | Africa OR „Subsaharan Africa” OR "West Africa" OR "Central Africa" OR "South Africa" OR "East Africa" OR "North Africa" OR Algieria OR Angola OR Benin OR Botswana OR „Burkina Faso” OR Burund OR Cameroon OR „Cape Verde” OR „Central African Republic” OR Chad OR Comoros OR Djibouti OR („DR Congo” OR „Democratic Republic of the Congo”) OR Egypt OR „Equatorial Guinea” OR Eritrea OR (Eswatini OR Swaziland) OR Ethiopia OR Gabon OR Gambia OR Ghana OR Guinea OR „Guinea-Bissau” OR („Ivory Coast” OR „Côte d'Ivoire") OR Kenya OR Lesotho OR Liberia OR Libya OR Madagascar OR Malawi OR Mali OR Mauritania OR Mauritius OR Morocco OR Mozambique OR Namibia OR Niger OR Nigeria OR „Republic of the Congo” OR Rwanda OR „São Tomé and Príncipe” OR Senegal OR Seychelles OR „Sierra Leone” OR Somalia OR „South Africa” OR „South Sudan” OR Sudan OR Tanzania OR Togo OR Tunisia OR Uganda OR „Western Sahara” OR Zambia OR Zimbabwe |

The generic search string was adapted to suit the constraints and functionalities of different databases. If possible, animal studies were excluded.

## **S1.2. Interpretation of MeSH Terms and Search Strategies**

To conduct a comprehensive literature review on the prevalence of filariasis in Africa from 2000 to 2025, the following MeSH terms and search strategies were employed:

**Filariasis:** "Filariasis" [MeSH Terms] OR "Filariasis" [All Fields] OR "Lymphatic Filariasis" [MeSH Terms] OR "Lymphatic Filariasis" [All Fields] OR "Onchocerciasis" [MeSH Terms] OR "Onchocerciasis" [All Fields] OR "Loiasis" [MeSH Terms] OR "Loiasis"[All Fields] OR "Mansonelliasis" [MeSH Terms] OR "Mansonelliasis" [All Fields] OR "Dirofilariasis" [MeSH Terms] OR "Dirofilariasis" [All Fields]

The use of MeSH terms ensures that the search encompasses articles indexed under these specific headings, while 'All Fields' broadens the search to include titles, abstracts, and other fields.

**Alternative terms** were added to account for alternative names and abbreviations that may be used in the literature: "elephantiasis" [All Fields] OR "river blindness" [All Fields] OR "african eye worm" [All Fields] OR "Mansonella spp." [All Fields] OR "Dirofilaria spp." [All Fields] OR "Wuchereria bancrofti" [All Fields] OR „W. bancrofti” [All Fields] OR "Brugia malayi" [All Fields] OR „B. malayi” [All Fields] OR "Onchocerca volvulus" [All Fields] OR „O. volvulus” [All Fields] OR "Loa loa" [All Fields] OR „L. loa” [All Fields].

**Keywords** for the identification of epidemiological studies: "Prevalence"[MeSH Terms] OR "Prevalence"[All Fields] OR "Incidence"[All Fields] OR "Epidemiology"[All Fields] OR "Occurrence" [All Fields] OR "Frequency"[All Fields]

**Countries and regions of Africa**: "Africa" OR "Subsaharan Africa" OR "West Africa" OR "Central Africa" OR "South Africa" OR "East Africa" OR "North Africa" OR "Algeria" OR "Angola" OR "Benin" OR "Botswana" OR "Burkina Faso" OR "Burundi" OR "Cameroon" OR "Cape Verde" OR "Central African Republic" OR "Chad" OR "Comoros" OR "Djibouti" OR ("DR Congo" OR "Democratic Republic of the Congo") OR "Egypt" OR "Equatorial Guinea" OR "Eritrea" OR ("Eswatini" OR "Swaziland") OR "Ethiopia" OR "Gabon" OR "Gambia" OR "Ghana" OR "Guinea" OR "Guinea-Bissau" OR ("Ivory Coast" OR "Côte d'Ivoire") OR "Kenya" OR "Lesotho" OR "Liberia" OR "Libya" OR "Madagascar" OR "Malawi" OR "Mali" OR "Mauritania" OR "Mauritius" OR "Morocco" OR "Mozambique" OR "Namibia" OR "Niger" OR "Nigeria" OR "Republic of the Congo" OR "Rwanda" OR "São Tomé and Príncipe" OR "Senegal" OR "Seychelles" OR "Sierra Leone" OR "Somalia" OR "South Africa" OR "South Sudan" OR "Sudan" OR "Tanzania" OR "Togo" OR "Tunisia" OR "Uganda" OR "Western Sahara" OR "Zambia" OR "Zimbabwe".

## **S1.3. Searches and Search Results (run 2025-10-08) for Each Database Below.**

**TableB .** Searches and search results for PubMed (2000 to October 2025).

| **#** | **Search terms** | **Results** |  |
| --- | --- | --- | --- |
| 1 | (Filariasis [MeSH Terms] OR "Lymphatic Filariasis" [MeSH Terms] OR Onchocerciasis [MeSH Terms] OR Loiasis [MeSH Terms] OR Mansonelliasis [MeSH Terms] OR Dirofilariasis [MeSH Terms] OR Elephantiasis OR "River blindness" OR "African eye worm" OR "*Mansonella* spp." OR "*Dirofilaria* spp." OR "*Wuchereria bancrofti*" OR "*W. bancrofti*" OR "*Brugia malayi*" OR "*B. malayi*" OR "*Onchocerca volvulus*" OR "*O. volvulus*" OR "*Loa loa*" OR "*L. loa*") | 20,324 |  |
| 2 | (Prevalence* [Title/Abstract] OR Incidence* [Title/Abstract] OR Epidemiology [MeSH Terms] OR Occurrence [Title/Abstract] OR Frequency [Title/Abstract]) | 3,292,330 |  |
| 3 | ("Africa" OR "Central Africa" OR "East Africa" OR "North Africa" OR "South Africa" OR "Subsaharan Africa" OR "West Africa" OR Algeri* OR Angol* OR Benin OR Botswan* OR "Burkina Faso" OR Burund* OR Cameroon* OR "Cape Verde" OR "Central African Republic" OR Chad OR Comoros OR Djibout* OR ("DR Congo" OR "Democratic Republic of the Congo") OR Egypt* OR "Equatorial Guinea" OR Eritre* OR ("Eswatini" OR Swaziland) OR Ethiop* OR Gabon OR Gambi* OR Ghan* OR Guine* OR "Guinea-Bissau" OR ("Ivory Coast" OR "Côte d'Ivoire") OR Keny* OR Lesoth* OR Liberi* OR Liby* OR Madagascar* OR Malaw* OR Mali OR Mauritani* OR Mauritius OR Morocco* OR Mozambiqu* OR Namibi* OR Niger* OR Nigeria* OR "Republic of the Congo" OR Rwand* OR "São Tomé and Príncipe" OR Senegal* OR Seychelles OR "Sierra Leone" OR Somali OR "South Sudan" OR Sudan* OR Tanzani* OR Togo OR Tunisi* OR Ugand* OR "Western Sahara" OR Zambi* OR Zimbabwe*) | 1,138,961 |  |
| 4 | 1 AND 2 AND 3 | 1,444 |  |
| 5 | 4 AND ("English"[Language]) AND ("2000"[Date - Publication] : "2025"[Date - Publication]) | 923 |  |
| 6 | 5 NOT ("Animals"[MeSH Terms]) | 89 |  |
| (Filariasis[MeSH Terms] OR "Lymphatic Filariasis"[MeSH Terms] OR Onchocerciasis[MeSH Terms] OR Loiasis[MeSH Terms] OR Mansonelliasis[MeSH Terms] OR Dirofilariasis[MeSH Terms] OR Elephantiasis OR "River blindness" OR "African eye worm" OR "*Mansonella* spp." OR "*Dirofilaria* spp." OR "*Wuchereria bancrofti*" OR "*W. bancrofti*" OR "*Brugia malayi*" OR "*B. malayi*" OR "*Onchocerca volvulus*" OR "*O. volvulus*" OR "*Loa loa*" OR "*L. loa*") AND (Prevalence*[Title/Abstract] OR Incidence*[Title/Abstract] OR Epidemiology[MeSH Terms] OR Occurrence[Title/Abstract] OR Frequency[Title/Abstract]) AND ("Africa" OR "Central Africa" OR "East Africa" OR "North Africa" OR "South Africa" OR "Subsaharan Africa" OR "West Africa" OR Algeri* OR Angol* OR Benin OR Botswan* OR "Burkina Faso" OR Burund* OR Cameroon* OR "Cape Verde" OR "Central African Republic" OR Chad OR Comoros OR Djibout* OR ("DR Congo" OR "Democratic Republic of the Congo") OR Egypt* OR "Equatorial Guinea" OR Eritre* OR ("Eswatini" OR Swaziland) OR Ethiop* OR Gabon OR Gambi* OR Ghan* OR Guine* OR "Guinea-Bissau" OR ("Ivory Coast" OR "Côte d'Ivoire") OR Keny* OR Lesoth* OR Liberi* OR Liby* OR Madagascar* OR Malaw* OR Mali OR Mauritani* OR Mauritius OR Morocco* OR Mozambiqu* OR Namibi* OR Niger* OR Nigeria* OR "Republic of the Congo" OR Rwand* OR "São Tomé and Príncipe" OR Senegal* OR Seychelles OR "Sierra Leone" OR Somali OR "South Sudan" OR Sudan* OR Tanzani* OR Togo OR Tunisi* OR Ugand* OR "Western Sahara" OR Zambi* OR Zimbabwe*) AND ("English"[Language] OR "French"[Language]) AND ("2000"[Date - Publication] : "2025"[Date - Publication]) NOT ("Animals"[MeSH Terms]) | | | |

**TableC .** Searches and search results for Scopus (2000 to October 2025).

| # | **Search terms** | Results |  |
| --- | --- | --- | --- |
| 1 | (Filariasis* OR "Lymphatic Filariasis" OR Onchocerciasis* OR Loiasis* OR Mansonelliasis* OR Dirofilariasis* OR Elephantiasis OR "River blindness" OR "African eye worm" OR "*Mansonella* spp." OR "*Dirofilaria* spp." OR "*Wuchereria bancrofti*" OR "*W. bancrofti*" OR "*Brugia malayi*" OR "*B. malayi*" OR "*Onchocerca volvulus*" OR "*O. volvulus*" OR "*Loa loa*" OR "*L. loa*") | 65,908 |  |
| 2 | (Prevalence* OR Incidence* OR Epidemiology* OR Occurrence OR Frequency) | 20,365,117 |  |
| 3 | ("Africa" OR "Central Africa" OR "East Africa" OR "North Africa" OR "South Africa" OR "Subsaharan Africa" OR "West Africa" OR Algeri* OR Angol* OR Benin OR Botswan* OR "Burkina Faso" OR Burund* OR Cameroon* OR "Cape Verde" OR "Central African Republic" OR Chad OR Comoros OR Djibout* OR ("DR Congo" OR "Democratic Republic of the Congo") OR Egypt* OR "Equatorial Guinea" OR Eritre* OR (Eswatini OR Swaziland) OR Ethiop* OR Gabon OR Gambi* OR Ghan* OR Guine* OR "Guinea-Bissau" OR ("Ivory Coast" OR "Côte d'Ivoire") OR Keny* OR Lesoth* OR Liberi* OR Liby* OR Madagascar* OR Malaw* OR Mali OR Mauritani* OR Mauritius OR Morocco* OR Mozambiqu* OR Namibi* OR Niger* OR Nigeria* OR "Republic of the Congo" OR Rwand* OR "São Tomé and Príncipe" OR Senegal* OR Seychelles OR "Sierra Leone" OR Somali OR "South Sudan" OR Sudan* OR Tanzani* OR Togo OR Tunisi* OR Ugand* OR "Western Sahara" OR Zambi* OR Zimbabwe*) | 9,575,483 |  |
| 4 | 1 AND 2 AND 3 | 20,186 |  |
| 5 | 4 AND (LIMIT-TO (PUBYEAR, 2000) OR LIMIT-TO (PUBYEAR, 2001) OR LIMIT-TO (PUBYEAR, 2002) OR LIMIT-TO (PUBYEAR, 2003) OR LIMIT-TO (PUBYEAR, 2004) OR LIMIT-TO (PUBYEAR, 2005) OR LIMIT-TO (PUBYEAR, 2006) OR LIMIT-TO (PUBYEAR, 2007) OR LIMIT-TO (PUBYEAR, 2008) OR LIMIT-TO (PUBYEAR, 2009) OR LIMIT-TO (PUBYEAR, 2010) OR LIMIT-TO (PUBYEAR, 2011) OR LIMIT-TO (PUBYEAR, 2012) OR LIMIT-TO (PUBYEAR, 2013) OR LIMIT-TO (PUBYEAR, 2014) OR LIMIT-TO (PUBYEAR, 2015) OR LIMIT-TO (PUBYEAR, 2016) OR LIMIT-TO (PUBYEAR, 2017) OR LIMIT-TO (PUBYEAR, 2018) OR LIMIT-TO (PUBYEAR, 2019) OR LIMIT-TO (PUBYEAR, 2020) OR LIMIT-TO (PUBYEAR,1 2021) OR LIMIT-TO (PUBYEAR, 2022) OR LIMIT-TO (PUBYEAR, 2023)2 OR LIMIT-TO (PUBYEAR, 2024) OR LIMIT-TO (PUBYEAR, 2025)) AND (LIMIT-TO (LANGUAGE, "English") | 14,937 |  |
| 6 | 5 AND (EXCLUDE (SUBJAREA, "MEDI") OR EXCLUDE (SUBJAREA, "VETE") | 4,304 |  |
| (Filariasis* OR "Lymphatic Filariasis" OR Onchocerciasis* OR Loiasis* OR Mansonelliasis* OR Dirofilariasis* OR Elephantiasis OR "River blindness" OR "African eye worm" OR "*Mansonella* spp." OR "*Dirofilaria* spp." OR "*Wuchereria bancrofti*" OR "*W. bancrofti*" OR "*Brugia malayi*" OR "*B. malayi*" OR "*Onchocerca volvulus*" OR "*O. volvulus*" OR "*Loa loa*" OR "*L. loa*") AND (prevalence* OR incidence* OR epidemiology* OR occurrence OR frequency) AND ("Africa" OR "Central Africa" OR "East Africa" OR "North Africa" OR "South Africa" OR "Subsaharan Africa" OR "West Africa" OR Algeri* OR Angol* OR Benin OR Botswan* OR "Burkina Faso" OR Burund* OR Cameroon* OR "Cape Verde" OR "Central African Republic" OR Chad OR Comoros OR Djibout* OR ("DR Congo" OR "Democratic Republic of the Congo") OR Egypt* OR "Equatorial Guinea" OR Eritre* OR (Eswatini OR Swaziland) OR Ethiop* OR Gabon OR Gambi* OR Ghan* OR Guine* OR "Guinea-Bissau" OR ("Ivory Coast" OR "Côte d'Ivoire") OR Keny* OR Lesoth* OR Liberi* OR Liby* OR Madagascar* OR Malaw* OR Mali OR Mauritani* OR Mauritius OR Morocco* OR Mozambiqu* OR Namibi* OR Niger* OR Nigeria* OR "Republic of the Congo" OR Rwand* OR "São Tomé and Príncipe" OR Senegal* OR Seychelles OR "Sierra Leone" OR Somali OR "South Sudan" OR Sudan* OR Tanzani* OR Togo OR Tunisi* OR Ugand* OR "Western Sahara" OR Zambi* OR Zimbabwe*) AND (LIMIT-TO (PUBYEAR, 2000) OR LIMIT-TO (PUBYEAR, 2001) OR LIMIT-TO (PUBYEAR, 2002) OR LIMIT-TO (PUBYEAR, 2003) OR LIMIT-TO (PUBYEAR, 2004) OR LIMIT-TO (PUBYEAR, 2005) OR LIMIT-TO (PUBYEAR, 2006) OR LIMIT-TO (PUBYEAR, 2007) OR LIMIT-TO (PUBYEAR, 2008) OR LIMIT-TO (PUBYEAR, 2009) OR LIMIT-TO (PUBYEAR, 2010) OR LIMIT-TO (PUBYEAR, 2011) OR LIMIT-TO (PUBYEAR, 2012) OR LIMIT-TO (PUBYEAR, 2013) OR LIMIT-TO (PUBYEAR, 2014) OR LIMIT-TO (PUBYEAR, 2015) OR LIMIT-TO (PUBYEAR, 2016) OR LIMIT-TO (PUBYEAR, 2017) OR LIMIT-TO (PUBYEAR, 2018) OR LIMIT-TO (PUBYEAR, 2019) OR LIMIT-TO (PUBYEAR, 2020) OR LIMIT-TO (PUBYEAR,1 2021) OR LIMIT-TO (PUBYEAR, 2022) OR LIMIT-TO (PUBYEAR, 2023)2 OR LIMIT-TO (PUBYEAR, 2024) OR LIMIT-TO (PUBYEAR, 2025)) AND (LIMIT-TO (LANGUAGE, "English")) AND (EXCLUDE (SUBJAREA, "MEDI") OR EXCLUDE (SUBJAREA, "VETE") | | | |

**Table D**Search and search results for ScienceDirect (2000 to October 2025).

| **#** | **Search terms** | **Results** |  |
| --- | --- | --- | --- |
| 1 | "Filariasis" OR "Onchocerciasis" OR "Loiasis" OR "Mansonelliasis" OR "Dirofilariasis" | 18,247 |  |
| 2 | "Angola" OR "Benin" OR "Botswana" OR "Burkina Faso" OR "Burundi" OR "Cameroon" OR "Cape Verde" OR "Central African Republic" OR "Chad" OR "Comoros" OR "Djibouti" OR "DR Congo" OR "Democratic Republic of the Congo" OR "Egypt" OR "Equatorial Guinea" OR "Eritrea" OR "Eswatini" OR "Swaziland" OR "Ethiopia" OR "Gabon" OR "Gambia" OR "Ghana" OR "Guinea" OR "Guinea-Bissau" OR "Ivory Coast" OR "Côte d'Ivoire" OR "Kenya" OR "Lesotho" OR "Liberia" OR "Libya" OR "Madagascar" OR "Malawi" OR "Mali" OR "Mauritania" OR "Mauritius" OR "Morocco" OR "Mozambique" OR "Namibia" OR "Niger" OR "Nigeria" OR "Republic of the Congo" OR "Réunion" OR "Rwanda" OR "São Tomé and Príncipe" OR "Senegal" OR "Seychelles" OR "Sierra Leone" OR "Somali" OR "South Sudan" OR "Sudan" OR "Tanzania" OR "Togo" OR "Tunisia" OR "Uganda" OR "Western Sahara" OR "Zambia" OR "Zimbabwe" | > 1,000,000 |  |
| 3 | “Filariasis” AND ("Angola" OR "Benin" OR "Bostawana" OR "Burkina Faso" OR "Burundi" OR "Cameroon" OR "Cape Verde" OR "Central African Republic") …  ("Onchocerciasis" OR "Loiasis" OR "Mansonelliasis" OR "Dirofilariasis") AND ("Angola" OR "Benin" OR "Bostawana" OR "Burkina Faso" OR "Burundi") …  FILTERS: Date: [2000-2025] AND "Research articles" AND "English" | 2920  2788 |  |
| Limit boolean connectors (AND, OR) = 8 | | | |

**TableE.** Searches and search results for AIM (African Index Medicus) (2000 to October 2025).

| **#** | **Search terms** | **Results** |  |
| --- | --- | --- | --- |
| 1 | ("Filariasis" OR "Lymphatic Filariasis" OR "Onchocerciasis" OR "Loiasis" OR "Mansonelliasis" OR "Dirofilariasis" OR "Elephantiasis" OR "River blindness" OR "African eye worm" OR "*Mansonella* spp." OR "*Dirofilaria* spp." OR "*Wuchereria bancrofti*" OR "*W. bancrofti*" OR "*Brugia malayi*" OR "*B. malayi*" OR "*Onchocerca volvulus*" OR "*O. volvulus*" OR "*Loa loa*" OR "*L. loa*") | 509 |  |
| 2 | ("Prevalence" OR "Incidence" OR "Epidemiology" OR "Occurrence" OR "Frequency") | 5,155 |  |
| 3 | "Africa" OR "Central Africa" OR "East Africa" OR "North Africa" OR "South Africa" OR "Subsaharan Africa" OR "West Africa" OR "Algeria" OR "Angola" OR "Benin" OR "Botswana" OR "Burkina Faso" OR "Burundi" OR "Cameroon" OR "Cape Verde" OR "Central African Republic" OR "Chad" OR "Comoros" OR "Djibouti" OR "DR Congo" OR "Democratic Republic of the Congo" OR "Egypt" OR "Equatorial Guinea" OR "Eritrea" OR "Eswatini" OR "Swaziland" OR "Ethiopia" OR "Gabon" OR "Gambia" OR "Ghana" OR "Guinea" OR "Guinea-Bissau" OR "Ivory Coast" OR "Côte d'Ivoire" OR "Kenya" OR "Lesotho" OR "Liberia" OR "Libya" OR "Madagascar" OR "Malawi" OR "Mali" OR "Mauritania" OR "Mauritius" OR "Morocco" OR "Mozambique" OR "Namibia" OR "Niger" OR "Nigeria" OR "Republic of the Congo" OR "Rwanda" OR "São Tomé and Príncipe" OR "Senegal" OR "Seychelles" OR "Sierra Leone" OR "Somali" OR "South Sudan" OR "Sudan" OR "Tanzania" OR "Togo" OR "Tunisia" OR "Uganda" OR "Western Sahara" OR "Zambia" OR "Zimbabwe" | 9.798 |  |
| 4 | 1 AND 2 AND 3 | 47 |  |
| 5 | 4 AND (2000...2025) | 21 |  |
| 6 | 5 AND ("English") | 21 |  |
| ("Filariasis" OR "Lymphatic Filariasis" OR "Onchocerciasis" OR "Loiasis" OR "Mansonelliasis" OR "Dirofilariasis" OR "Elephantiasis" OR "River blindness" OR "African eye worm" OR "*Mansonella* spp." OR "*Dirofilaria* spp." OR "*Wuchereria bancrofti*" OR "*W. bancrofti*" OR "*Brugia malayi*" OR "*B. malayi*" OR "*Onchocerca volvulus*" OR "*O. volvulus*" OR "*Loa loa*" OR "*L. loa*") AND ("Prevalence" OR "Incidence" OR "Epidemiology" OR "Occurrence" OR "Frequency") AND ("Africa" OR "Central Africa" OR "East Africa" OR "North Africa" OR "South Africa" OR "Subsaharan Africa" OR "West Africa" OR "Algeria" OR "Angola" OR "Benin" OR "Botswana" OR "Burkina Faso" OR "Burundi" OR "Cameroon" OR "Cape Verde" OR "Central African Republic" OR "Chad" OR "Comoros" OR "Djibouti" OR "DR Congo" OR "Democratic Republic of the Congo" OR "Egypt" OR "Equatorial Guinea" OR "Eritrea" OR "Eswatini" OR "Swaziland" OR "Ethiopia" OR "Gabon" OR "Gambia" OR "Ghana" OR "Guinea" OR "Guinea-Bissau" OR "Ivory Coast" OR "Côte d'Ivoire" OR "Kenya" OR "Lesotho" OR "Liberia" OR "Libya" OR "Madagascar" OR "Malawi" OR "Mali" OR "Mauritania" OR "Mauritius" OR "Morocco" OR "Mozambique" OR "Namibia" OR "Niger" OR "Nigeria" OR "Republic of the Congo" OR "Rwanda" OR "São Tomé and Príncipe" OR "Senegal" OR "Seychelles" OR "Sierra Leone" OR "Somali" OR "South Sudan" OR "Sudan" OR "Tanzania" OR "Togo" OR "Tunisia" OR "Uganda" OR "Western Sahara" OR "Zambia" OR "Zimbabwe") AND (2000...2025) AND ("English") | | | |
