# Supplementary material for: Human Filariasis in Africa (2000–2025): Changing epidemiology, uneven diagnostic progress, and persistent neglect
Source: PLoS Negl Trop Dis. 2026 Apr 7;20(4):e0014200. doi: 10.1371/journal.pntd.0014200 (PMC13082706; doi:10.1371/journal.pntd.0014200)
Supplement: S3 Appendix — (DOCX) [file pntd.0014200.s003.docx]

# **S3 Appendix**

Quality of the publications included in this systematic review was assessed using the Newcastle–Ottawa Scale (NOS), adapted for cross-sectional prevalence studies.

Three main domains were evaluated: selection (maximum 4 points), comparability (maximum 2 points), and outcome assessment (maximum 3 points), for a total of 9 possible points.

## **Selection**

**Clearly defined study population** – 1 point was assigned if authors reported key demographic and environmental variables (age, sex, place of residence, and living conditions such as access to water or sanitation).

**Sample representativeness** – 1 point was assigned if samples were randomly selected or otherwise representative, with potential selection bias addressed and an adequate sample size reported.

**Clearly stated inclusion and exclusion criteria** – 1 point was assigned when both were explicitly defined.

**Validated diagnostic method** – 1 point was assigned when a standardized and validated diagnostic technique (microscopy, serology, or molecular) was used.

## **Comparability**

**Control for confounding factors** – 1 point was assigned when authors accounted for key confounders (e.g., age, sex, sanitation, access to water).

**Consistency of diagnostic methods** – 1 point was assigned when comparable diagnostic techniques were used across study groups.

## **Outcome assessment**

**Objective measurement of infection prevalence** – 1 point was assigned when assessments were conducted by trained personnel using pre-defined diagnostic protocols.

**Appropriate statistical analysis** – 1 point was assigned when statistical methods were clearly described and suitable for prevalence estimation.

**Reporting of data loss or non-response** – 1 point was assigned if data loss or response rate was reported and its potential impact on bias discussed.

Based on the total NOS score, studies were classified as:

High risk of bias: ≤5 points

Moderate risk: 6–7 points

Low risk: 8–9 points

**Note:** The scale was adapted to reflect characteristics of epidemiological studies of filarial infections in Africa, where field-based sampling and variable diagnostic tools are common.

**Table A**. Quality assessment and risk of bias evaluation using the Newcastle-Ottawa Scale.

| First author | Year | Selection (maximum of 4 stars) | Comparability (maximum of  2 stars) | Outcome (maximum of 3 stars) | Total Score | | Quality | | Ref. |
| --- | --- | --- | --- | --- | --- | --- | --- | --- | --- |
| Abd-Elfarag et al. | 2020 | *** | ** | ** | | 7 | Good | 1 | |
| Aboagye-Antwi et al. | 2015 | *** | ** | * | | 6 | Fair | 2 | |
| Adekunle 2016 et al. | 2016 | *** | ** | ** | | 7 | Good | 3 | |
| Adekunle, et al. | 2018 | *** | ** | ** | | 7 | Good | 4 | |
| Adu Mensah et al. | 2022 | *** | ** | ** | | 7 | Good | 5 | |
| Agbolade et al. | 2001 | *** | * | ** | | 6 | Fair | 6 | |
| Akafyi et al. | 2015 | *** | ** | ** | | 7 | Good | 7 | |
| Akue et al. | 2011 | *** | ** | ** | | 7 | Good | 8 | |
| Anagbogu et al. | 2022 | *** | * | ** | | 6 | Fair | 9 | |
| Anosike et al. | 2004 | *** | * | * | | 5 | Fair | 10 | |
| Ashton  et al. | 2011 | *** | ** | ** | | 7 | Good | 11 | |
| Ayong  et al. | 2005 | *** | ** | ** | | 7 | Good | 12 | |
| Aza'ah et al. | 2020 | *** | ** | * | | 6 | Fair | 13 | |
| Badaki et al. | 2013 | *** | ** | ** | | 7 | Good | 14 | |
| Bah et al. | 2020 | *** | ** | * | | 6 | Fair | 15 | |
| Bakajika et al. | 2014 | *** | ** | ** | | 7 | Good | 16 | |
| Bassene et al. | 2015 | *** | ** | *** | | 8 | Good | 17 | |
| Beng et al. | 2020 | *** | ** | *** | | 8 | Good | 18 | |
| Bhwana et al. | 2022 | *** | ** | * | | 6 | Fair | 19 | |
| Bienvenu Nwane et al. | 2025 | *** | * | ** | | 6 | Fair | 20 | |
| Boateng  et al. | 2025 | *** | ** | ** | | 7 | Good | 21 | |
| Boko-Collins et al. | 2019 | **** | ** | ** | | 8 | Good | 22 | |
| Boullé  et al. | 2019 | *** | ** | * | | 6 | Fair | 23 | |
| Brant et al. | 2017 | *** | ** | * | | 6 | Fair | 24 | |
| Campillo et al. | 2024 | *** | * | ** | | 6 | Fair | 25 | |
| Campillo et al. | 2023 | *** | ** | ** | | 7 | Good | 26 | |
| Chesnais et al. | 2019 | *** | ** | ** | | 7 | Good | 27 | |
| Chesnais et al. | 2014 | *** | ** | ** | | 7 | Good | 28 | |
| Cho-Ngwa  et al. | 2009 | *** | ** | ** | | 7 | Good | 29 | |
| Christiana  et al. | 2014 | *** | * | *** | | 7 | Good | 30 | |
| Coalson  et al. | 2024 | *** | ** | ** | | 7 | Good | 31 | |
| Coulibaly et al. | 2016 | *** | ** | * | | 6 | Fair | 32 | |
| Coulibaly et al. | 2023 | *** | ** | *** | | 8 | Good | 33 | |
| Coulibaly et al. | 2015 | **** | ** | ** | | 8 | Good | 34 | |
| Coulibaly et al. | 2021 | **** | ** | ** | | 8 | Good | 35 | |
| Dana et al. | 2015 | *** | ** | ** | | 7 | Good |  | |
|  |  |  |  |  | |  |  |  | |
| de Smet et al. | 2020 | *** | ** | ** | | 7 | Good | 37 | |
| de Souza  et al. | 2015 | *** | ** | ** | | 7 | Good | 38 | |
| Debrah et al. | 2017 | *** | ** | *** | | 8 | Good | 39 | |
| Dogara et al. | 2012 | *** | * | * | | 5 | Fair | 40 | |
| Dolo et al. | 2018 | *** | * | *** | | 7 | Good | 41 | |
| Dolo et al. | 2023 | *** | ** | ** | | 7 | Good | 42 | |
| Dolo et al. | 2019 | *** | ** | ** | | 7 | Good | 43 | |
| Dorkenoo et al. | 2018 | *** | ** | * | | 6 | Fair | 44 | |
| Dorkenoo et al. | 2021 | *** | ** | ** | | 7 | Good | 45 | |
| Drame et al. | 2016 | *** | ** | *** | | 8 | Good | 46 | |
| Ekpo et al. | 2022 | ** | * | ** | | 5 | Fair | 47 | |
| Ella et al. | 2021 | *** | ** | *** | | 8 | Good | 48 | |
| Emukah  et al. | 2018 | *** | ** | ** | | 7 | Good | 49 | |
| Endeshaw et al. | 2015 | *** | ** | ** | | 7 | Good | 50 | |
| Engelbrecht et al. | 2003 | *** | ** | * | | 6 | Fair | 51 | |
| Evans et al. | 2014 | *** | ** | ** | | 7 | Good | 52 | |
| Forrer et al. | 2021 | *** | ** | *** | | 8 | Good | 53 | |
| Garchitorena et al. | 2018 | *** | ** | ** | | 7 | Good | 54 | |
| Gbakima et al. | 2005 | *** | ** | * | | 6 | Fair | 55 | |
| Gebrezgabiher et al. | 2020 | *** | ** | ** | | 7 | Good | 56 | |
| Golden et al. | 2016 | *** | * | ** | | 6 | Fair | 57 | |
| Guiguemde et al. | 2020 | *** | ** | ** | | 7 | Good | 58 | |
| Hadermann | 2025 | *** | ** | ** | | 7 | Good | 59 | |
| Hassan et al. | 2011 | *** | ** | *** | | 8 | Good | 60 | |
| Hassen et al. | 2023 | *** | ** | ** | | 7 | Good | 61 | |
| Hernández-González et al. | 2016 | *** | ** | ** | | 7 | Good | 62 | |
| Hildebrandt et al. | 2024 | *** | ** | *** | | 8 | Good | 63 | |
| Iboh et al. | 2012 | *** | ** | *** | | 8 | Good | 64 | |
| Ityonzughul et al. | 2024 | *** | ** | * | | 6 | Fair | 65 | |
| Ivoke et al. | 2015 | *** | ** | ** | | 7 | Good | 66 | |
| Jones et al. | 2018 | *** | ** | ** | | 7 | Good | 67 | |
| Jones et al. | 2017 | *** | ** | ** | | 7 | Good | 68 | |
| Kamga et al. | 2011 | *** | ** | ** | | 7 | Good | 69 | |
| Kamga  et al. | 2016 | *** | ** | ** | | 7 | Good | 70 | |
| Kamgno et al. | 2017 | *** | ** | *** | | 8 | Good | 71 | |
| Kamtchum Tatuene et al. | 2014 | *** | * | ** | | 6 | Fair | 72 | |
| Kargbo-Labour et al. | 2024 | *** | * | *** | | 7 | Good | 73 | |
| Katabarwa et al. | 2008 | *** | ** | * | | 6 | Fair | 74 | |
| Katabarwa et al. | 2011 | *** | ** | ** | | 7 | Good | 75 | |
| Katabarwa et al. | 2010 | *** | ** | ** | | 7 | Good | 76 | |
| Katabarwa et al. | 2016 | *** | ** | ** | | 7 | Good | 77 | |
| Katabarwa et al. | 2013 | *** | ** | ** | | 7 | Good | 78 | |
| Kima et al. | 2021 | **** | ** | ** | | 8 | Good | 79 | |
| King  et al. | 2012 | *** | ** | * | | 6 | Fair | 80 | |
| Kinyatta et al. | 2023 | *** | ** | ** | | 7 | Good | 81 | |
|  |  |  |  |  | |  |  |  | |
| Koala et al. | 2017 | **** | * | ** | | 7 | Good | 82 | |
| Komlan et al. | 2018 | *** | ** | *** | | 8 | Good | 83 | |
| Korbmacher et al. | 2018 | *** | ** | ** | | 7 | Good | 84 | |
| Koroma et al. | 2013 | *** | ** | * | | 6 | Fair | 85 | |
| Koroma et al. | 2018 | *** | ** | ** | | 7 | Good | 86 | |
| Koroma et al. | 2018 | *** | ** | ** | | 7 | Good | 87 | |
| Koroma et al. | 2012 | *** | ** | ** | | 7 | Good | 88 | |
| Kouassi et al. | 2015 | *** | ** | * | | 6 | Fair | 89 | |
| Kroidl et al. | 2016 | *** | ** | * | | 6 | Fair | 90 | |
| Ladan et al. | 2018 | *** | ** | ** | | 7 | Good | 91 | |
| Lakwo et al. | 2020 | *** | ** | ** | | 7 | Good | 92 | |
| Lenaerts et al. | 2018 | *** | * | ** | | 6 | Fair | 93 | |
| Lloyd  et al. | 2015 | *** | ** | ** | | 7 | Good | 94 | |
| Lupenza et al. | 2022 | **** | ** | ** | | 8 | Good | 95 | |
| Luroni et al. | 2017 | *** | ** | ** | | 7 | Good | 96 | |
| Mahenge et al. | 2022 | *** | ** | ** | | 7 | Good | 97 | |
| MalhotraI et al. | 2003 | *** | ** | ** | | 7 | Good | 98 | |
| M'Bondoukwé et al. | 2024 | *** | ** | * | | 6 | Fair | 99 | |
| M'bondoukwé et al. | 2018 | *** | ** | ** | | 7 | Good | 100 | |
| Midzi et al. | 2025 | **** | ** | ** | | 8 | Good | 101 | |
| Minetti et al. | 2019 | *** | ** | * | | 6 | Fair | 102 | |
| Mnkai et al. | 2022 | *** | ** | ** | | 7 | Good | 103 | |
| Mountongo et al. | 2023 | *** | * | ** | | 6 | Fair | 104 | |
| Mourembou et al. | 2015 | *** | ** | ** | | 7 | Good | 104 | |
| Moustafa et al. | 2014 | *** | ** | ** | | 7 | Good | 105 | |
| Moutongo Mouandza et al. | 2024 | *** | ** | ** | | 7 | Good | 106 | |
| Moya et al. | 2016 | *** | ** | * | | 6 | Fair | 107 | |
| Msyamboza  et al. | 2010 | *** | ** | * | | 6 | Fair | 108 | |
| Mukoko et al. | 2004 | **** | ** | * | | 7 | Good | 109 | |
| Murdoch  et al. | 2017 | *** | ** | * | | 6 | Fair | 110 | |
| Mushi et al. | 2024 | *** | ** | *** | | 8 | Good | 111 | |
| Mwakitalu et al. | 2013 | *** | ** | ** | | 7 | Good | 112 | |
| Mwakitalu et al. | 2013 | **** | ** | ** | | 8 | Good | 113 | |
| Mwase et al. | 2014 | *** | ** | ** | | 8 | Good | 114 | |
| Mwesigye et al. | 2024 | *** | ** | ** | | 7 | Good | 115 | |
| Mweya  et al. | 2007 | *** | ** | ** | | 7 | Good | 116 | |
| Nana-Djeunga et al. | 2022 | *** | ** | ** | | 7 | Good | 117 | |
| Nana-Djeunga et al. | 2017 | *** | ** | ** | | 7 | Good | 118 | |
| Nana-Djeunga et al. | 2015 | *** | ** | *** | | 8 | Good | 119 | |
| Ncogo et al. | 2024 | *** | ** | *** | | 8 | Good | 120 | |
| Nditanchou et al. | 2023 | *** | ** | * | | 6 | Fair | 121 | |
| Ngwira et al. | 2007 | **** | ** | * | | 7 | Good | 122 | |
| Nchang et al. | 2025 | *** | ** | ** | | 7 | Good | 123 | |
| Nikièma et al. | 2024 | *** | ** | *** | | 8 | Good | 124 | |
| Njamnshi et al. | 2024 | *** | ** | *** | | 8 | Good | 125 | |
| Njenga et al. | 2011 | **** | ** | ** | | 8 | Good | 126 | |
| Nsakashalo-Senkwe et al. | 2017 | **** | ** | * | | 7 | Good | 127 | |
| Nyagang et al. | 2020 | *** | ** | ** | | 7 | Good | 128 | |
| OgouyÃ, et al. | 2017 | *** | ** | ** | | 7 | Good | 129 | |
| Oguttu et al. | 2014 | *** | ** | ** | | 7 | Good | 130 | |
| Ojurongbe et al. | 2015 | *** | * | *** | | 7 | Good | 131 | |
| Okon et al. | 2010 | *** | ** | ** | | 7 | Good | 132 | |
| Okorie et al. | 2015 | *** | ** | * | | 6 | Fair | 133 | |
| Omudu et al. | 2011 | **** | ** | ** | | 8 | Good | 134 | |
| Opare et al. | 2025 | *** | ** | * | | 6 | Fair | 135 | |
| Opoku et al. | 2024 | ** | * | ** | | 5 | Fair | 136 | |
| Otabil et al. | 2023 | *** | ** | *** | | 8 | Good | 137 | |
| Otabil et al. | 2019 | *** | ** | *** | | 8 | Good | 138 | |
| Ouedraogo et al. | 2016 | *** | ** | ** | | 7 | Good | 139 | |
| Ouedraogo et al. | 2024 | *** | ** | ** | | 7 | Good | 140 | |
| Pam et al. | 2017 | *** | ** | ** | | 7 | Good | 141 | |
| Parkouda et al. | 2024 | ** | * | ** | | 5 | Fair | 142 | |
| Paulo et al. | 2020 | *** | ** | ** | | 7 | Good | 143 | |
| Pion SDS et al. | 2016 | *** | ** | ** | | 7 | Good | 144 | |
| Pion SDS et al. | 2017 | *** | ** | * | | 6 | Fair | 145 | |
| Pion SDS et al. | 2020 | *** | ** | ** | | 7 | Good | 146 | |
| Rakers et al. | 2020 | ** | ** | ** | | 6 | Fair | 147 | |
| Rebollo et al. | 2015 | *** | ** | * | | 6 | Fair | 148 | |
| Ruberanziza et al. | 2009 | *** | ** | ** | | 7 | Good | 149 | |
| Sam-Wobo  et al. | 2012 | *** | ** | * | | 6 | Fair | 150 | |
| Sandri et al. | 2021 | *** | ** | ** | | 7 | Good | 151 | |
| Sangare et al. | 2018 | *** | ** | ** | | 7 | Good | 152 | |
|  |  |  |  |  | |  |  |  | |
| Senkwe  et al. | 2022 | *** | ** | ** | | 7 | Good | 153 | |
| Shawa  et al. | 2013 | *** | ** | ** | | 7 | Good | 154 | |
| Siewe et al. | 2019 | *** | ** | ** | | 7 | Good | 155 | |
| Siewe Fodjo et al. | 2022 | *** | ** | *** | | 8 | Good | 156 | |
| Siewe Fodjo et al. | 2021 | *** | ** | *** | | 8 | Good | 157 | |
| Siewe Fodjo et al. | 2018 | *** | ** | *** | | 8 | Good | 158 | |
| Siewe Fodjo 2019 et al. | 2019 | *** | ** | ** | | 7 | Good | 159 | |
| Sumo et al. | 2022 | *** | ** | *** | | 8 | Good | 160 | |
| Surakat et al. | 2023 | *** | ** | *** | | 8 | Good | 161 | |
| Ta TH et al. | 2018 | *** | ** | ** | | 7 | Good | 162 | |
| Tafatatha et al. | 2015 | *** | ** | * | | 6 | Fair | 163 | |
| Tekle  et al. | 2012 | *** | ** | ** | | 7 | Good | 164 | |
|  |  |  |  |  | |  |  |  | |
| Trevisan et al. | 2025 | *** | ** | *** | | 8 | Good | 165 | |
| Tsapi et al. | 2020 | **** | ** | ** | | 8 | Good | 166 | |
| Uttah et al. | 2011 | *** | * | * | | 5 | Fair | 167 | |
| Uttah et al. | 2011 | *** | ** | ** | | 7 | Good | 168 | |
| Van-Dúnem et al. | 2023 | *** | * | *** | | 7 | Good | 169 | |
| Van-Dúnem et al. | 2024 | **** | * | ** | | 7 | Good | 170 | |
| Veletzky et al. | 2022 | *** | ** | *** | | 8 | Good | 171 | |
| Wanji et al. | 2015 | *** | * | ** | | 6 | Fair | 172 | |
| Wanji et al. | 2012 | *** | ** | ** | | 7 | Good | 173 | |
| Wanji et al. | 2015 | *** | ** | ** | | 7 | Good | 174 | |
| Wanji et al. | 2015 | *** | ** | ** | | 7 | Good | 175 | |
| Wanji  et al. | 2016 | *** | ** | * | | 6 | Fair | 176 | |
| Wilson et al. | 2016 | *** | ** | *** | | 8 | Good | 177 | |
| Won et al. | 2018 | *** | ** | ** | | 7 | Good | 178 | |
| Yaya et al. | 2014 | *** | * | * | | 5 | Fair | 179 | |
| Yokoly et al. | 2020 | *** | ** | *** | | 8 | Good | 180 | |

* Indicates one criteria was followed, ** two criteria were followed, ***three criteria were followed, and **** four criteria were followed

Reference

**1.** Abd-Elfarag G, Carter JY, Raimon S, Sebit W, Suliman A, Fodjo JNS, et al. Persons with onchocerciasis-associated epilepsy and nodding seizures have a more severe form of epilepsy with more cognitive impairment and higher levels of Onchocerca volvulus infection. Epileptic Disord. 2020;22:301–308. doi:10.1684/epd.2020.1164.

**2.** Aboagye-Antwi F, Kwansa-Bentum B, Dadzie SK, Ahorlu CK, Appawu MA, Gyapong J, et al. Transmission indices and microfilariae prevalence in human population prior to mass drug administration with ivermectin and albendazole in the Gomoa District of Ghana. Parasit Vectors. 2015;8:562. doi:10.1186/s13071-015-1105-x.

**3.** Adekunle NO, Sam-Wobo SO, Adeleke MA, Ekpo UF, Davies E, Ladokun AO, et al. Prevalence and distribution of Wuchereria bancrofti in Ose local government area, Ondo state, Nigeria. Niger J Parasitol. 2016;37:96–100. doi:10.4314/njpar.v37i1.19.

**4.** Adekunle NO, Asimiea AO. Prevalence of lymphatic filariasis and associated clinical morbidities among adolescents in three rural communities in Ondo State, Southwest Nigeria. J Trop Med Health. 2018;2. doi:10.29011/JTMH-120.000120.

5. Adu Mensah D, Debrah LB, Gyamfi PA, Rahamani AA, et al. Occurrence of lymphatic filariasis infection after 15 years of mass drug administration in two hotspot districts in the Upper East Region of Ghana. *PLoS Negl Trop Dis*. 2022;16(8):e0010129. doi:10.1371/journal.pntd.0010129.

**6.** Agbolade M, Akinboye DO. Loa loa and Mansonella perstans infections in Ijebu North, western Nigeria: a parasitological study. Jpn J Infect Dis. 2001;54(3):108–110. PMID:11544400.

**7.** Akafyi DE, Oko JO, Bonire FS, Elkanah OS, Abdullahi M. Clinical and parasitological assessment of lymphatic filariasis in Jahum Local Government Area, Jigawa State. J Microbial Biotech Res. 2015;5:25–31.

**8.** Akue JP, Nkoghe D, Padilla C, Moussavou G, Moukana H, Mbou RA, et al. Epidemiology of concomitant infection due to Loa loa and Mansonella perstans in Gabon. PLoS Negl Trop Dis. 2011;5:e1329. doi:10.1371/journal.pntd.0001329.

**9.** Anagbogu IN, Saka YA, Surakat OA, Okoronkwo C, Davies E, Oyale P, et al. Integrated transmission assessment surveys (iTAS) of lymphatic filariasis and onchocerciasis in Cross River, Taraba and Yobe States, Nigeria. Parasit Vectors. 2022;15:201. doi:10.1186/s13071-022-05302-x.

**10.** Anosike JC, Nwoke BEB, Onwuliri COE, Obiukwu CE, Duru AF, Nwachukwu MI, et al. Prevalence of parasitic diseases among nomadic Fulanis of south-eastern Nigeria. Ann Agric Environ Med. 2004;11(2):221–225. PMID:15627328.

**11.** Ashton RA, Kyabayinze DJ, Opio T, Auma A, Edwards T, Matwale G, et al. The impact of mass drug administration and long-lasting insecticidal net distribution on Wuchereria bancrofti infection in humans and mosquitoes: an observational study in northern Uganda. Parasit Vectors. 2011;4:134. doi:10.1186/1756-3305-4-134.

**12.** Ayong LS, Tume CB, Wembe FE, Simo G, Asonganyi T, Lando G, et al. Development and evaluation of an antigen detection dipstick assay for the diagnosis of human onchocerciasis. Trop Med Int Health. 2005;10:228–233. doi:10.1111/j.1365-3156.2004.01384.x.

**13.** Aza’ah RA, Sumo L, Ntonifor NH, Bopda J, Bamou RH, Nana-Djeunga HC. Point prevalence mapping reveals hotspot for onchocerciasis transmission in the Ndikinimeki Health District, Centre Region, Cameroon. Parasit Vectors. 2020;13:519. doi:10.1186/s13071-020-04387-6.

**14.** Badaki JA, Akogun OB, Molta NB, Imandeh G. Bancroftiasis among the Mumuye of Northeastern Nigeria: parasitological and clinical studies in Northern Taraba State. Health. 2013;5:138–142. doi:10.4236/health.2013.51018.

**15.** Bah YM, Paye J, Bah MS, Conteh A, Redwood-Sawyerr V, Sonnie M, et al. Achievements and challenges of lymphatic filariasis elimination in Sierra Leone. PLoS Negl Trop Dis. 2020;14:e0008877. doi:10.1371/journal.pntd.0008877.

**16.** Bakajika DK, Nigo MM, Lotsima JP, Masikini GA, Fischer K, Lloyd MM, et al. Filarial antigenemia and Loa loa night blood microfilaremia in an area without bancroftian filariasis in the Democratic Republic of Congo. Am J Trop Med Hyg. 2014;91:1142–1148. doi:10.4269/ajtmh.14-0358.

**17.** Bassene H, Sambou M, Fenollar F, Clarke S, Djiba S, Mourembou G, et al. High prevalence of Mansonella perstans filariasis in rural Senegal. Am J Trop Med Hyg. 2015;93:601–606. doi:10.4269/ajtmh.15-0051.

**18.** Beng AA, Esum ME, Deribe K, Njouendou AJ, Ndongmo PWC, Abong RA, et al. Mapping lymphatic filariasis in Loa loa endemic health districts naïve for ivermectin mass administration and situated in the forested zone of Cameroon. BMC Infect Dis. 2020;20:284. doi:10.1186/s12879-020-05009-3.

**19.** Bhwana D, Mmbando BP, Dusabimana A, Mhina A, Challe DP, Fodjo JNS, et al. Ivermectin treatment response in two rural villages with a high prevalence of onchocerciasis and epilepsy, Mahenge Tanzania. Afr Health Sci. 2022;22:607–616. doi:10.4314/ahs.v22i3.65.

**20.** Bienvenu Nwane P, Nana-Djeunga HC, Toche NN, Domché A, Bertrand FN, Niamsi YE, et al. Status of human onchocerciasis transmission in the Adamaoua region of Cameroon after 20 years of ivermectin mass distribution. PLoS Negl Trop Dis. 2025;19:e0011511. doi:10.1371/journal.pntd.0011511.

**21.** Boateng CA, Afatodzie MS, McLure A, Kwansa-Bentum B, de Souza DK. Lymphatic filariasis transmission 10 years after stopping mass drug administration in the Gomoa West District of Ghana. Int J Infect Dis. 2025;152:107790. doi:10.1016/j.ijid.2025.107790.

**22.** Boko-Collins PM, Ogouyemi-Hounto A, Adjinacou-Badou EG, Gbaguidi-Saizonou L, Dossa NI, Dare A, et al. Assessment of treatment impact on lymphatic filariasis in 13 districts of Benin: progress toward elimination in nine districts despite persistence of transmission in some areas. Parasites Vectors. 2019;12:276. doi:10.1186/s13071-019-3525-5.

**23.** Boullé C, Njamnshi AK, Dema F, Mengnjo MK, Siewe Fodjo JN, Bissek A-CZ-K, et al. Impact of 19 years of mass drug administration with ivermectin on epilepsy burden in a hyperendemic onchocerciasis area in Cameroon. Parasit Vectors. 2019;12:114. doi:10.1186/s13071-019-3345-7.

**24.** Brant TA, Okorie PN, Ogunmola O, Ojeyode NB, Fatunade SB, Davies E, et al. Integrated risk mapping and landscape characterisation of lymphatic filariasis and loiasis in South West Nigeria. Parasite Epidemiol Control. 2018;3:21–35. doi:10.1016/j.parepi.2017.12.001.

**25.** Campillo JT, Hemilembolo MC, Pion SDS, Lebredonchel E, Dupasquier V, Boullé C, et al. Association between blood Loa loa microfilarial density and proteinuria levels in a rural area of the Republic of Congo (the MorLo project): a population-based cross-sectional study. Lancet Microbe. 2023;4:e704–e710. doi:10.1016/S2666-5247(23)00142-8.

**26.** Campillo JT, Biamonte MA, Hemilembolo MC, Missamou F, Boussinesq M, Pion SDS, et al. Evaluation of a novel biplex rapid diagnostic test for antibody responses to Loa loa and Onchocerca volvulus infections. PLoS Negl Trop Dis. 2024;18:e0012567. doi:10.1371/journal.pntd.0012567.

**27.** Chesnais CB, Missamou F, Pion SD, Bopda J, Louya F, Majewski AC, et al. A case study of risk factors for lymphatic filariasis in the Republic of Congo. Parasit Vectors. 2014;7:300. doi:10.1186/1756-3305-7-300.

**28.** Chesnais CB, Awaca-Uvon N-P, Vlaminck J, Tambwe J-P, Weil GJ, Pion SD, et al. Risk factors for lymphatic filariasis in two villages of the Democratic Republic of the Congo. Parasit Vectors. 2019;12:162. doi:10.1186/s13071-019-3428-5.

**29.** Cho-Ngwa F, Amambua AN, Ambele MA, Titanji VPK. Evidence for the exacerbation of lymphedema of geochemical origin, podoconiosis, by onchocerciasis. J Infect Public Health. 2009;2:198–203. doi:10.1016/j.jiph.2009.09.006.

**30.** Christiana O, Olajumoke M, Oyetunde S. Lymphatic filariasis and associated morbidities in rural communities of Ogun State, Southwestern Nigeria. Travel Med Infect Dis. 2014;12:95–101. doi:10.1016/j.tmaid.2013.02.006.

**31.** Coalson JE, Noland GS, Nute AW, Goodhew EB, Martin DL, Abdalla Z, et al. Integrated serosurveillance for onchocerciasis, lymphatic filariasis, and schistosomiasis in North Darfur, Sudan. Am J Trop Med Hyg. 2024;111:58–68. doi:10.4269/ajtmh.23-0760.

**32.** Coulibaly YI, Dembele B, Diallo AA, Konaté S, Dolo H, Coulibaly SY, et al. The impact of six annual rounds of mass drug administration on Wuchereria bancrofti infections in humans and in mosquitoes in Mali. Am J Trop Med Hyg. 2015;93:356–360. doi:10.4269/ajtmh.14-0516.

**33.** Coulibaly YI, Coulibaly SY, Dolo H, Konate S, Diallo AA, Doumbia SS, et al. Dynamics of antigenemia and transmission intensity of Wuchereria bancrofti following cessation of mass drug administration in a formerly highly endemic region of Mali. Parasit Vectors. 2016;9:628. doi:10.1186/s13071-016-1911-9.

**34.** Coulibaly S, Sawadogo S, Hien A, Nikièma A, Sangare I, Bamogo R, et al. Malaria and lymphatic filariasis co-transmission in endemic health districts in Burkina Faso. Adv Entomol. 2021;09:155–175. doi:10.4236/ae.2021.94014.

**35.** Coulibaly YI, Sangare M, Dolo H, Soumaoro L, Coulibaly SY, Dicko I, et al. No evidence of lymphatic filariasis transmission in Bamako urban setting after three mass drug administration rounds. Parasitol Res. 2022;121:3243–3248. doi:10.1007/s00436-022-07648-8.

**36.** Dana D, Debalke S, Mekonnen Z, Kassahun W, Suleman S, Getahun K, et al. A community-based cross-sectional study of the epidemiology of onchocerciasis in unmapped villages for community-directed treatment with ivermectin in Jimma Zone, southwestern Ethiopia. BMC Public Health. 2015;15:595. doi:10.1186/s12889-015-1888-x.

**37.** de Smet E, Metanmo S, Mbelesso P, Kemata B, Siewe Fodjo JN, Boumédiène F, et al. Focus of ongoing onchocerciasis transmission close to Bangui, Central African Republic. Pathogens. 2020;9:337. doi:10.3390/pathogens9050337.

**38.** de Souza DK, Ansumana R, Sessay S, Conteh A, Koudou B, Rebollo MP, et al. The impact of residual infections on Anopheles-transmitted Wuchereria bancrofti after multiple rounds of mass drug administration. Parasit Vectors. 2015;8:488. doi:10.1186/s13071-015-1091-z.

**39.** Debrah LB, Nausch N, Opoku VS, Owusu W, Mubarik Y, Berko DA, et al. Epidemiology of Mansonella perstans in the middle belt of Ghana. Parasit Vectors. 2017;10:15. doi:10.1186/s13071-016-1960-0.

**40.** Dogara MM, Nock HI, Agbede RIS, Ndams S, Joseph K. Prevalence of lymphatic filariasis in three villages in Kano State, Nigeria. Int J Trop Med. 2012;8:1–8.

**41.** Dolo H, Coulibaly YI, Kelly-Hope L, Konate S, Dembele B, Coulibaly SY, et al. Factors associated with Wuchereria bancrofti microfilaremia in an endemic area of Mali. Am J Trop Med Hyg. 2018;98:1782–1787. doi:10.4269/ajtmh.17-0902.

42. Dolo H, Coulibaly ME, Sow M, Coulibaly YI, Doumbia M, Sangare M, et al. Progress towards elimination of onchocerciasis transmission in Mali: a “pre-stop MDA” survey in 18 transmission zones. *PLoS Negl Trop Dis.* 2023;17:e0011632. doi:10.1371/journal.pntd.0011632.

43. Dolo H, Coulibaly YI, Dembele B, Guindo B, Coulibaly SY, Dicko I, et al. Integrated seroprevalence-based assessment of *Wuchereria bancrofti* and *Onchocerca volvulus* in two lymphatic filariasis evaluation units of Mali with the SD Bioline Onchocerciasis/LF IgG4 Rapid Test. *PLoS Negl Trop Dis.* 2019;13:e0007064. doi:10.1371/journal.pntd.0007064.

44. Dorkenoo MA, Bronzan R, Yehadji D, Tchalim M, Yakpa K, Etassoli S, et al. Surveillance for lymphatic filariasis after stopping mass drug administration in endemic districts of Togo, 2010–2015. *Parasit Vectors.* 2018;11:244. doi:10.1186/s13071-018-2843-3.

45. Dorkenoo AM, Koba A, Halatoko WA, Teko M, Kossi K, Yakpa K, et al. Assessment of the usefulness of anti-Wb123 antibody for post-elimination surveillance of lymphatic filariasis. *Parasit Vectors.* 2021;14:23. doi:10.1186/s13071-020-04535-y.

46. Drame PM, Montavon C, Pion SD, Kubofcik J, Fay MP, Nutman TB. Molecular epidemiology of blood-borne human parasites in a *Loa loa*, *Mansonella perstans*, and *Plasmodium falciparum* endemic region of Cameroon. *Am J Trop Med Hyg.* 2016;94:1301–1308. doi:10.4269/ajtmh.15-0746.

47. Ekpo UF, Eneanya OA, Nwankwo EN, Soneye IY, Weil GJ, Fischer PU, et al. Persistence of onchocerciasis in villages in Enugu and Ogun states in Nigeria following many rounds of mass distribution of ivermectin. *BMC Infect Dis.* 2022;22:832. doi:10.1186/s12879-022-07811-7.

48. Ella SN, Ogoussan K, Gass K, Hundley L, Diggle PJ, Johnson O, et al. An integrated district mapping strategy for loiasis to enable safe mass treatment for onchocerciasis in Gabon. *Am J Trop Med Hyg.* 2021;106:732–739. doi:10.4269/ajtmh.21-0799.

49. Emukah E, Rakers LJ, Kahansim B, Miri ES, Nwoke BEB, Griswold E, et al. In southern Nigeria, *Loa loa* blood microfilaria density is very low even in areas with high prevalence of loiasis: results of a survey using the new LoaScope technology. *Am J Trop Med Hyg.* 2018;99:116–123. doi:10.4269/ajtmh.18-0163.

50. Endeshaw T, Taye A, Tadesse Z, Katabarwa MN, Shafi O, Seid T, et al. Presence of *Wuchereria bancrofti* microfilaremia despite 7 years of annual ivermectin monotherapy mass drug administration for onchocerciasis control: a study in north-west Ethiopia. *Pathog Glob Health.* 2015;109:344–351. doi:10.1080/20477724.2015.1103501.

51. Engelbrecht F, Oettl T, Herter U, Link C, Philipp D, Edeghere H, et al. Analysis of *Wuchereria bancrofti* infections in a village community in northern Nigeria: increased prevalence in individuals infected with *Onchocerca volvulus*. *Parasitol Int.* 2003;52:13–20. doi:10.1016/s1383-5769(02)00045-4.

52. Evans DS, Alphonsus K, Umaru J, Eigege A, Miri E, Mafuyai H, et al. Status of onchocerciasis transmission after more than a decade of mass drug administration for onchocerciasis and lymphatic filariasis elimination in central Nigeria: challenges in coordinating the stop MDA decision. *PLoS Negl Trop Dis.* 2014;8:e3113. doi:10.1371/journal.pntd.0003113.

53. Forrer A, Wanji S, Obie ED, Nji TM, Hamill L, Ozano K, et al. Why onchocerciasis transmission persists after 15 annual ivermectin mass drug administrations in South-West Cameroon. *BMJ Glob Health.* 2021;6. doi:10.1136/bmjgh-2020-003248.

54. Garchitorena A, Raza-Fanomezanjanahary EM, Mioramalala SA, Chesnais CB, Ratsimbasoa CA, Ramarosata H, et al. Towards elimination of lymphatic filariasis in southeastern Madagascar: successes and challenges for interrupting transmission. *PLoS Negl Trop Dis.* 2018;12:e0006780. doi:10.1371/journal.pntd.0006780.

55. Gbakima AA, Appawu MA, Dadzie S, Karikari C, Sackey SO, Baffoe-Wilmot A, et al. Lymphatic filariasis in Ghana: establishing the potential for an urban cycle of transmission. *Trop Med Int Health.* 2005;10:387–392. doi:10.1111/j.1365-3156.2005.01389.x.

56. Gebrezgabiher G, Mekonnen Z, Yewhalaw D, Hailu A. Status of parasitological indicators and morbidity burden of onchocerciasis after years of successive implementation of mass distribution of ivermectin in selected communities of Yeki and Asosa districts, Ethiopia. *BMC Public Health.* 2020;20:1233. doi:10.1186/s12889-020-09344-7.

57. Golden A, Faulx D, Kalnoky M, Stevens E, Yokobe L, Peck R, et al. Analysis of age-dependent trends in Ov16 IgG4 seroprevalence to onchocerciasis. *Parasit Vectors.* 2016;9:338. doi:10.1186/s13071-016-1623-1.

58. Guiguemde KT, Sawadogo PM, Zida A, Kima A, Bougma RW, Serme M, et al. Situation of onchocerciasis transmission in 2020 in the Cascades region of Burkina Faso. *Parasitology Int.* 2024;98:102822. doi:10.1016/j.parint.2023.102822.

59. Hadermann A, Jada SR, Lubbers C, Amaral LJ, Biamonte M, de Souza DK, Bol YY, Siewe Fodjo JN, Colebunders R. A novel biplex *Onchocerca volvulus* rapid diagnostic test evaluated among 3- to 9-year-old children in Maridi, South Sudan. *Diagnostics (Basel).* 2025;15(5):563. doi:10.3390/diagnostics15050563. PMID:40075810; PMCID:PMC11898602.

60. Hassan AA, Akinsanya B, Iyase N, Owagboriaye FO. Assessment of loiasis and outcomes of ivermectin mass treatment in Ijebu-North, Nigeria. *Korean J Parasitol.* 2011;49:153–159. doi:10.3347/kjp.2011.49.2.153.

61. Hassen M, Mohammed A, Endeshaw T, Seid T, Samuel F, Asmare T, et al. Integrated prevalence assessment of *Wuchereria bancrofti* and *Onchocerca volvulus* in three co-endemic districts of Gambella Region, Ethiopia. *Am J Trop Med Hyg.* 2023;109:844–849. doi:10.4269/ajtmh.22-0392.

**62.** Hernández-González A, Moya L, Perteguer MJ, Herrador Z, Nguema R, Nguema J, et al. Evaluation of onchocerciasis seroprevalence in Bioko Island (Equatorial Guinea) after years of disease control programmes. Parasit Vectors. 2016;9:509. doi:10.1186/s13071-016-1779-8.

**63.** Hildebrandt TR, Davi SD, Kabwende AL, Endamne LR, Mehmel E, Rakotonirinalalao M, et al. Evaluation of knowledge, attitude and practices towards loiasis in the rural community of Sindara, in central African Gabon. PLoS Negl Trop Dis. 2024;18:e0012109. doi:10.1371/journal.pntd.0012109.

**64.** Iboh CI, Okon OE, Opara KN, Asor JE, Etim SE. Lymphatic filariasis among the Yakurr people of Cross River State, Nigeria. Parasit Vectors. 2012;5:203. doi:10.1186/1756-3305-5-203.

**65.** Ityonzughul C, Sallau A, Miri E, Emukah E, Kahansim B, Adelamo S, et al. The interruption of transmission of onchocerciasis in Abia, Anambra, Enugu, and Imo States, Nigeria: the largest global onchocerciasis stop-treatment decision to date. Pathogens. 2024;13. doi:10.3390/pathogens13080671.

**66.** Ivoke N, Ezeabikwa BO, Ivoke ON, Ekeh FN, Ezenwaji NE, Odo GE, et al. Wuchereria bancrofti infection in rural tropical guinea savannah communities: rapid epidemiological assessment using immunochromatographic card test and prevalence of hydrocoele. Trop Biomed. 2015;32(2):365–375.

**67.** Jones C, Ngasalla B, Derua YA, Tarimo D, Malecela MN. Lymphatic filariasis elimination efforts in Rufiji, southeastern Tanzania: decline in circulating filarial antigen prevalence in young school children after twelve rounds of mass drug administration and utilization of long-lasting insecticide-treated nets. Int J Infect Dis. 2017;61:38–43. doi:10.1016/j.ijid.2017.05.009.

**68.** Jones C, Ngasala B, Derua YA, Tarimo D, Reimer L, Bockarie M, et al. Lymphatic filariasis transmission in Rufiji District, southeastern Tanzania: infection status of the human population and mosquito vectors after twelve rounds of mass drug administration. Parasit Vectors. 2018;11:588. doi:10.1186/s13071-018-3156-2.

**69.** Kamga HLF, Shey DN, Assob JCN, Njunda AL, Nde Fon P, Njem PK. Prevalence of onchocerciasis in the Fundong Health District, Cameroon after 6 years of continuous community-directed treatment with ivermectin. Pan Afr Med J. 2011;10:34.

**70.** Kamga G-R, Dissak-Delon FN, Nana-Djeunga HC, Biholong BD, Mbigha-Ghogomu S, Souopgui J, et al. Still mesoendemic onchocerciasis in two Cameroonian community-directed treatment with ivermectin projects despite more than 15 years of mass treatment. Parasit Vectors. 2016;9:581. doi:10.1186/s13071-016-1868-8.

**71.** Kamgno J, Pion SD, Chesnais CB, Bakalar MH, D’Ambrosio MV, Mackenzie CD, et al. A test-and-not-treat strategy for onchocerciasis in Loa loa-endemic areas. N Engl J Med. 2017;377:2044–2052. doi:10.1056/NEJMoa1705026.

72. Kamtchum Tatuene J, Gounoue R, Nkoa T, Tchatchueng Mbougua J, Nana Djeunga HC, Bopda J, et al. Epidemiology of *Loa loa* and *Mansonella perstans* filariasis in the Akonolinga health district, Centre Region, Cameroon. *Health Sci. Dis.* 2014;15:1–5.

73. Kargbo-Labour I, Bah MS, Melchers NVSV, Conteh A, Redwood-Sawyerr V, Stolk WA, et al. Impact assessment of onchocerciasis through lymphatic filariasis transmission assessment surveys using Ov-16 rapid diagnostic tests in Sierra Leone. *Parasit Vectors.* 2024;17:121. doi:10.1186/s13071-024-06198-5.

74. Katabarwa M, Eyamba A, Habomugisha P, Lakwo T, Ekobo S, Kamgno J, et al. After a decade of annual dose mass ivermectin treatment in Cameroon and Uganda, onchocerciasis transmission continues. *Trop Med Int Health.* 2008;13:1196–1203. doi:10.1111/j.1365-3156.2008.02126.x.

75. Katabarwa MN, Eyamba A, Chouaibou M, Enyong P, Kuété T, Yaya S, et al. Does onchocerciasis transmission take place in hypoendemic areas? A study from the North Region of Cameroon. *Trop Med Int Health.* 2010;15:645–652. doi:10.1111/j.1365-3156.2010.02501.x.

76. Katabarwa MN, Eyamba A, Nwane P, Enyong P, Yaya S, Baldiagaï J, et al. Seventeen years of annual distribution of ivermectin has not interrupted onchocerciasis transmission in North Region, Cameroon. *Am J Trop Med Hyg.* 2011;85:1041–1049. doi:10.4269/ajtmh.2011.11-0333.

77. Katabarwa MN, Lakwo T, Habomugisha P, Agunyo S, Byamukama E, Oguttu D, et al. Transmission of *Onchocerca volvulus* continues in Nyagak-Bondo focus of northwestern Uganda after 18 years of a single dose of annual treatment with ivermectin. *Am J Trop Med Hyg.* 2013;89:293–300. doi:10.4269/ajtmh.13-0037.

78. Katabarwa MN, Katamanywa J, Lakwo T, Habomugisha P, Byamukama E, Oguttu D, et al. The Imaramagambo onchocerciasis focus in Southwestern Uganda: interruption of transmission after disappearance of the vector *Simulium neavei* and its associated freshwater crabs. *Am J Trop Med Hyg.* 2016;95:417–425. doi:10.4269/ajtmh.16-0181.

79. Kima A, Guiguemde KT, Serme M, Meda ZC, Bougma R, Djiatsa JP, et al. Lymphatic filariasis transmission assessment survey in Burkina Faso in connection with 4 districts. *Med Trop Sante Int.* 2021;1:mtsibulletin.n1.2021.83. doi:10.48327/mtsibulletin.n1.2021.83.

80. King JD, Eigege A, Umaru J, Jip N, Miri E, Jiya J, et al. Evidence for stopping mass drug administration for lymphatic filariasis in some, but not all local government areas of Plateau and Nasarawa States, Nigeria. *Am J Trop Med Hyg.* 2012;87:272–280. doi:10.4269/ajtmh.2012.11-0718.

81. Kinyatta N, Wachira D, Githae R, Lusweti J, Ingonga J, Ichugu C, et al. Detection of *Wuchereria bancrofti* in human blood samples and mosquitoes in Matayos, Busia County-Kenya. *Sci Rep.* 2023;13:19420. doi:10.1038/s41598-023-46329-z.

82. Koala L, Nikiema A, Post RJ, Paré AB, Kafando CM, Drabo F, et al. Recrudescence of onchocerciasis in the Comoé valley in Southwest Burkina Faso. *Acta Trop.* 2017;166:96–105. doi:10.1016/j.actatropica.2016.11.003.

83. Komlan K, Vossberg PS, Gantin RG, Solim T, Korbmacher F, Banla M, et al. *Onchocerca volvulus* infection and serological prevalence, ocular onchocerciasis and parasite transmission in northern and central Togo after decades of *Simulium damnosum* s.l. vector control and mass drug administration of ivermectin. *PLoS Negl Trop Dis.* 2018;12:e0006312. doi:10.1371/journal.pntd.0006312.

84. Korbmacher F, Komlan K, Gantin RG, Poutouli WP, Padjoudoum K, Karabou P, et al. *Mansonella perstans, Onchocerca volvulus* and *Strongyloides stercoralis* infections in rural populations in central and southern Togo. *Parasite Epidemiol Control.* 2018;3:77–87. doi:10.1016/j.parepi.2018.03.001.

85. Koroma JB, Bangura MM, Hodges MH, Bah MS, Zhang Y, Bockarie MJ. Lymphatic filariasis mapping by immunochromatographic test cards and baseline microfilaria survey prior to mass drug administration in Sierra Leone. *Parasit Vectors.* 2012;5:10. doi:10.1186/1756-3305-5-10.

86. Koroma JB, Sesay S, Sonnie M, Hodges MH, Sahr F, Zhang Y, et al. Impact of three rounds of mass drug administration on lymphatic filariasis in areas previously treated for onchocerciasis in Sierra Leone. *PLoS Negl Trop Dis.* 2013;7:e2273. doi:10.1371/journal.pntd.0002273.

87. Koroma JB, Sesay S, Conteh A, Paye J, Bah M, Sonnie M, et al. Progress on elimination of lymphatic filariasis in Sierra Leone. *Parasit Vectors.* 2018;11:334. doi:10.1186/s13071-018-2915-4.

88. Koroma JB, Sesay S, Conteh A, Koudou B, Paye J, Bah M, et al. Impact of five annual rounds of mass drug administration with ivermectin on onchocerciasis in Sierra Leone. *Infect Dis Poverty.* 2018;7:30. doi:10.1186/s40249-018-0410-y.

89. Kouassi BL, de Souza DK, Goepogui A, Narh CA, King SA, Mamadou BS, et al. Assessing the presence of *Wuchereria bancrofti* in vector and human populations from urban communities in Conakry, Guinea. *Parasit Vectors.* 2015;8:492. doi:10.1186/s13071-015-1077-x.

90. Kroidl I, Saathof E, Maganga L, Clowes P, Maboko L, Hoerauf A, et al. Prevalence of lymphatic filariasis and treatment effectiveness of albendazole/ivermectin in individuals with HIV co-infection in Southwest-Tanzania. *PLoS Negl Trop Dis.* 2016;10:e0004618. doi:10.1371/journal.pntd.0004618.

91. Ladan MU, Tukur AT, Moyi SI. Seroprevalence of lymphatic filariasis in six communities of Bungudu Local Government Area, Zamfara State, Nigeria. *Int J Pure Appl Biosci.* 2018;6:11–18. doi:10.18782/2320-7051.6568.

92. Lakwo TL, Raimon S, Tionga M, Siewe Fodjo JN, Alinda P, Sebit WJ, et al. The role of the Maridi aam in causing an onchocerciasis-associated epilepsy epidemic in Maridi, South Sudan: an epidemiological, sociological, and entomological study. *Pathogens.* 2020;9. doi:10.3390/pathogens9040315.

93. Lenaerts E, Mandro M, Mukendi D, Suykerbuyk P, Dolo H, Wonya’Rossi D, et al. High prevalence of epilepsy in onchocerciasis endemic health areas in Democratic Republic of the Congo. *Infect Dis Poverty.* 2018;7:68. doi:10.1186/s40249-018-0452-1.

94. Lloyd MM, Gilbert R, Taha NT, Weil GJ, Meite A, Kouakou IMM, et al. Conventional parasitology and DNA-based diagnostic methods for onchocerciasis elimination programmes. *Acta Trop.* 2015;146:114–118. doi:10.1016/j.actatropica.2015.03.019.

95. Lupenza ET, Gasarasi DB, Minzi OM. Lymphatic filariasis elimination status: *Wuchereria bancrofti* infections in human populations and factors contributing to continued transmission after seven rounds of mass drug administration in Masasi District, Tanzania. *PLoS One.* 2022;17:e0262693. doi:10.1371/journal.pone.0262693.

96. Luroni LT, Gabriel M, Tukahebwa E, Onapa AW, Tinkitina B, Tukesiga E, et al. The interruption of *Onchocerca volvulus* and *Wuchereria bancrofti* transmission by integrated chemotherapy in the Obongi focus, North Western Uganda. *PLoS One.* 2017;12:e0189306. doi:10.1371/journal.pone.0189306.

97. M’bondoukwé NP, Kendjo E, Mawili-Mboumba DP, Koumba Lengongo JV, Offouga Mbouoronde C, Nkoghe D, et al. Prevalence of and risk factors for malaria, filariasis, and intestinal parasites as single infections or co-infections in different settlements of Gabon, Central Africa. *Infect Dis Poverty.* 2018;7:6. doi:10.1186/s40249-017-0381-4.

98. M’Bondoukwé NP, Owono-Medang M, Moussavou-Boussougou MN, Akoue Y, Migueba V, Bulaev D, et al. Low diagnostic performance of thick blood smears of 50 µl in comparison with direct examination of 10 µl blood and the leukoconcentration technique of 5 ml blood among loiasis-suspected patients with low microfilaremia in Gabon, Central Africa, using the STARD-BLCM guidelines. *Parasit Vectors.* 2024;17:138. doi:10.1186/s13071-023-06089-1.

99. Malhotra I, Ouma JH, Wamachi A, Kioko J, Mungai P, Njzovu M, et al. Influence of maternal filariasis on childhood infection and immunity to *Wuchereria bancrofti* in Kenya. *Infect Immun.* 2003;71:5231–5237. doi:10.1128/iai.71.9.5231-5237.2003.

100. Midzi N, Mutsaka-Makuvaza MJ, Phiri I, Palatio K, Bakajika D, Zouré HM, et al. Shrinking the lymphatic filariasis map of Zimbabwe: reassessing the population requiring treatment through confirmatory mapping. *Int J Infect Dis.* 2025;152:107791. doi:10.1016/j.ijid.2025.107791.

101. Minetti C, Tettevi EJ, Mechan F, Prada JM, Idun B, Biritwum N-K, et al. Elimination within reach: a cross-sectional study highlighting the factors that contribute to persistent lymphatic filariasis in eight communities in rural Ghana. *PLoS Negl Trop Dis.* 2019;13:e0006994. doi:10.1371/journal.pntd.0006994.

102. Mnkai J, Marandu TF, Mhidze J, Urio A, Maganga L, Haule A, et al. Step towards elimination of *Wuchereria bancrofti* in Southwest Tanzania 10 years after mass drug administration with albendazole and ivermectin. *PLoS Negl Trop Dis.* 2022;16:e0010044. doi:10.1371/journal.pntd.0010044.

103. Mourembou G, Fenollar F, Lekana-Douki JB, Ndjoyi Mbiguino A, Maghendji Nzondo S, Matsiegui PB, et al. *Mansonella*, including a potential new species, as common parasites in children in Gabon. *PLoS Negl Trop Dis.* 2015;9:e0004155. doi:10.1371/journal.pntd.0004155.

104. Moustafa MA, Thabet HS, Saad GA, El-Setouhy M, Mehrez M, Hamdy DM. Surveillance of lymphatic filariasis 5 years after stopping mass drug administration in Menoufiya Governorate, Egypt. *East Mediterr Health J.* 2014;20:295–299.

105. Moutongo Mouandza R, Mourou JR, Moutombi Ditombi B, Roger Sibi Matotou H, Ekomi B, Bouyou-Akotet MK, et al. Sociodemographics, clinical factors, and biological factors associated with loiasis in endemic onchocerciasis areas in Southern Gabon. *Am J Trop Med Hyg.* 2023;109:850–857. doi:10.4269/ajtmh.22-0558.

106. Moutongo Mouandza R, Mourou Mbina JR, Moutombi Ditombi B, Mihindou JC, Moussavou Mabicka DA, Mayandza C, et al. Prevalence and sociodemographic risk factors of soil-transmitted helminths in rural communities living in endemic foci of onchocerciasis in Southern Gabon. *Pathogens.* 2024;13. doi:10.3390/pathogens13110967.

107. Moya L, Herrador Z, Ta-Tang TH, Rubio JM, Perteguer MJ, Hernandez-González A, et al. Evidence for suppression of onchocerciasis transmission in Bioko Island, Equatorial Guinea. *PLoS Negl Trop Dis.* 2016;10:e0004829. doi:10.1371/journal.pntd.0004829.

108. Msyamboza K, Ngwira B, Banda R, Mkwanda S, Brabin B. Sentinel surveillance of lymphatic filariasis, schistosomiasis, soil-transmitted helminths and malaria in rural southern Malawi. *Malawi Med J.* 2010;22:12–14. doi:10.4314/mmj.v22i1.55901.

109. Mukoko DAN, Pedersen EM, Masese NN, Estambale BBA, JHO. Bancroftian filariasis in 12 villages in Kwale district, Coast province, Kenya — variation in clinical and parasitological patterns. *Ann Trop Med Parasitol.* 2004;98:801–815. doi:10.1179/000349804X3225.

110. Murdoch ME, Murdoch IE, Evans J, Yahaya H, Njepuome N, Cousens S, et al. Pre-control relationship of onchocercal skin disease with onchocercal infection in Guinea Savanna, Northern Nigeria. *PLoS Negl Trop Dis.* 2017;11:e0005489. doi:10.1371/journal.pntd.0005489.

111. Mushi VP, Bhwana D, Massawe IS, Makunde W, Sebukoto H, Ngasa W, et al. Prevalence of onchocerciasis and epilepsy in a Tanzanian region after a prolonged community-directed treatment with ivermectin. *PLoS Negl Trop Dis.* 2024;18:e0012470. doi:10.1371/journal.pntd.0012470.

112. Mwakitalu ME, Malecela MN, Pedersen EM, Mosha FW, Simonsen PE. Urban lymphatic filariasis in the metropolis of Dar es Salaam, Tanzania. *Parasit Vectors.* 2013;6:286. doi:10.1186/1756-3305-6-286.

113. Mwakitalu ME, Malecela MN, Pedersen EM, Mosha FW, Simonsen PE. Urban lymphatic filariasis in the city of Tanga, Tanzania, after seven rounds of mass drug administration. *Acta Trop.* 2013;128:692–700. doi:10.1016/j.actatropica.2013.10.004.

114. Mwase ET, Stensgaard A-S, Nsakashalo-Senkwe M, Mubila L, Mwansa J, Songolo P, et al. Mapping the geographical distribution of lymphatic filariasis in Zambia. *PLoS Negl Trop Dis.* 2014;8:e2714. doi:10.1371/journal.pntd.0002714.

115. Mwesigye V, Musinguzi B, Okongo B, Mucunguzi W, Kakaire MN, Migisha R. Sero-antigen prevalence of lymphatic filariasis and risk factors of podoconiosis in Busiriba sub-county, Kamwenge district, Southwestern Uganda, August–September 2018. *BMC Res Notes.* 2024;17:141. doi:10.1186/s13104-024-06801-z.

116. Mweya CN, Kalinga AK, Kabula B, Malley KD, Ruhiso MH, Maegga BTA. Onchocerciasis situation in the Tukuyu focus of southwest Tanzania after ten years of ivermectin mass treatment. *Tanzan Health Res Bull.* 2007;9:174–179. doi:10.4314/thrb.v9i3.14325.

117. Nana-Djeunga HC, Tchatchueng-Mbougua JB, Bopda J, Mbickmen-Tchana S, Elong-Kana N, Nnomzo’o E, et al. Mapping of bancroftian filariasis in Cameroon: prospects for elimination. *PLoS Negl Trop Dis.* 2015;9:1–19. doi:10.1371/journal.pntd.0004001.

118. Nana-Djeunga HC, Tchouakui M, Njitchouang GR, Tchatchueng-Mbougua JB, Nwane P, Domche A, et al. First evidence of lymphatic filariasis transmission interruption in Cameroon: progress towards elimination. *PLoS Negl Trop Dis.* 2017;11:e0005633. doi:10.1371/journal.pntd.0005633.

119. Nana-Djeunga HC, Djune-Yemeli L, Domche A, Donfo-Azafack C, Efon-Ekangouo A, Lenou-Nanga C, et al. High infection rates for onchocerciasis and soil-transmitted helminthiasis in children under five not receiving preventive chemotherapy: a bottleneck to elimination. *Infect Dis Poverty.* 2022;11:47. doi:10.1186/s40249-022-00973-1.

120. Nchang LC, Magha C, Fonong PA, Gandjui NVT, Tchatat NM, Nkimbeng DA, Nietcho FN, Foyet JV, Fombad FF, Katcho TD, Cho JF, Hoerauf A, Ritter M, Wanji S. Parasitic infection prevalence in tuberculosis patients and their household contacts in the Littoral Region of Cameroon. *Parasite Epidemiol Control.* 2025;28:e00409. doi:10.1016/j.parepi.2025.e00409. PMID:39867582; PMCID:PMC7617333.

121. Ncogo P, Hernández-González A, Ta-Tang T-H, Redondo L, Álvarez A, Perteguer MJ, et al. Approaching onchocerciasis elimination in Equatorial Guinea: near zero transmission and public health implication. *Infect Dis Poverty.* 2024;13:86. doi:10.1186/s40249-024-01254-9.

**122.** Nditanchou R, Dixon R, Atekem K, Biholong B, Wilhelm A, Selby R, et al. Ivermectin and doxycycline treatments against onchocerciasis: adaptations and impact among semi-nomadic population in Massangam Health District, Cameroon. PLoS Negl Trop Dis. 2023;17:e0011463. doi:10.1371/journal.pntd.0011463.

**123.** Ngwira BM, Tambala P, Perez AM, Bowie C, Molyneux DH. The geographical distribution of lymphatic filariasis infection in Malawi. Filaria J. 2007;6:12. doi:10.1186/1475-2883-6-12.

124. Nikièma AS, Koala L, Post RJ, Kima A, et al. Progress towards elimination of onchocerciasis in the Region du Sud-Ouest of Burkina Faso which was previously subject to a recrudescence event after vector control. *PLoS Negl Trop Dis*. 2024;18(4):e0012118. doi:10.1371/journal.pntd.0012118. PMID:38683750; PMCID:PMC11057763.

**125.** Njamnshi WY, Siewe Fodjo JN, Njamnshi KG, Ngarka L, Mengnjo MK, Nfor LN, et al. Dementia prevalence and Onchocerca volvulus infection among rural elderly persons in the Ntui Health District, Cameroon: a population-based study. Pathogens. 2024;13. doi:10.3390/pathogens13070568.

**126.** Njenga SM, Mwandawiro CS, Wamae CN, Mukoko DA, Omar AA, Shimada M, et al. Sustained reduction in prevalence of lymphatic filariasis infection in spite of missed rounds of mass drug administration in an area under mosquito nets for malaria control. Parasit Vectors. 2011;4:90. doi:10.1186/1756-3305-4-90.

**127.** Nsakashalo-Senkwe M, Mwase E, Chizema-Kawesha E, Mukonka V, Songolo P, Masaninga F, et al. Significant decline in lymphatic filariasis associated with nationwide scale-up of insecticide-treated nets in Zambia. Parasite Epidemiol Control. 2017;2:7–14. doi:10.1016/j.parepi.2017.08.001.

**128.** Nyagang SM, Cumber SN, Cho JF, Keka EI, Nkfusai CN, Wepngong E, et al. Prevalence of onchocerciasis, attitudes and practices and the treatment coverage after 15 years of mass drug administration with ivermectin in the Tombel Health District, Cameroon. Pan Afr Med J. 2020;35:107. doi:10.11604/pamj.2020.35.107.16036.

**129.** Ogouyá A, Ibikounle M, de Tove YSS, Dare A, Batcho W, Kabore A. Success of lymphatic filariasis control in Benin: effects of ivermectin and albendazole on microfilaraemia. J Parasitol Vector Biol. 2017;9:19–26. doi:10.5897/JPVB2016.0280.

**130.** Oguttu D, Byamukama E, Katholi CR, Habomugisha P, Nahabwe C, Ngabirano M, et al. Serosurveillance to monitor onchocerciasis elimination: the Ugandan experience. Am J Trop Med Hyg. 2014;90:339–345. doi:10.4269/ajtmh.13-0546.

**131.** Ojurongbe O, Akindele AA, Adeleke MA, Oyedeji MO, Adedokun SA, Ojo JF, et al. Co-endemicity of loiasis and onchocerciasis in rain forest communities in southwestern Nigeria. PLoS Negl Trop Dis. 2015;9:e0003633. doi:10.1371/journal.pntd.0003633.

**132.** Okon OE, Iboh CI, Opara KN. Bancroftian filariasis among the Mbembe people of Cross River state, Nigeria. J Vector Borne Dis. 2010;47:91–96.

**133.** Okorie PN, Davies E, Ogunmola OO, Ojurongbe O, Saka Y, Okoeguale B, et al. Lymphatic filariasis baseline survey in two sentinel sites of Ogun state, Nigeria. Pan Afr Med J. 2015;20:397. doi:10.11604/pamj.2015.20.397.5686.

**134.** Omudu EA, Ochoga JO. Clinical epidemiology of lymphatic filariasis and community practices and perceptions amongst the Ado people of Benue state, Nigeria. Afr J Infect Dis. 2011;5:47–53.

**135.** Opare JL, de Souza DK, Alomatu B, Mensah E, Nyarko E, Asiedu O, et al. Confirmatory mapping for lymphatic filariasis in districts previously considered nonendemic in Ghana. Int J Infect Dis. 2025;152:107801. doi:10.1016/j.ijid.2025.107801.

136. Opoku M, de Souza DK. Identification and characterisation of *Mansonella perstans* in the Volta Region of Ghana. *PLoS ONE*. 2024;19(6):e0295089. doi:10.1371/journal.pone.0295089.

**137.** Otabil KB, Gyasi SF, Awuah E, Obeng-Ofori D, Atta-Nyarko RJ, Andoh D, et al. Prevalence of onchocerciasis and associated clinical manifestations in selected hypoendemic communities in Ghana following long-term administration of ivermectin. BMC Infect Dis. 2019;19:431. doi:10.1186/s12879-019-4076-2.

**138.** Otabil KB, Basáñez M-G, Ankrah B, Opoku SA, Kyei DO, Hagan R, et al. Persistence of onchocerciasis and associated dermatologic and ophthalmic pathologies after 27 years of ivermectin mass drug administration in the middle belt of Ghana. Trop Med Int Health. 2023;28:844–854. doi:10.1111/tmi.13937.

**139.** Ouedraogo AN, Somda EB, Traoré F, Ouédraogo MS, Tapsoba GP, Ouangre A, et al. Impact du traitement de masse de la filariose lymphatique par l’albendazole-ivermectine en zone de savane: cas de la région de l’Est du Burkina. Health Sci Dis. 2016;17. doi:10.5281/hsd.v17i4.731.

**140.** Ouedraogo MO, Meda IB, Kourouma K, Wienne FY, Nare D, Bougouma C, et al. Effects of five years of treatment of onchocerciasis with ivermectin under community guidelines in resurgent areas of Burkina Faso: a before-and-after analysis. Trop Med Infect Dis. 2024;9. doi:10.3390/tropicalmed9090207.

**141.** Pam DD, de Souza DK, D’Souza S, Opoku M, Sanda S, Nazaradden I, et al. Is mass drug administration against lymphatic filariasis required in urban settings? The experience in Kano, Nigeria. PLoS Negl Trop Dis. 2017;11:e0006004. doi:10.1371/journal.pntd.0006004.

**142.** Parkouda S, Saidou M, Bisseye C. Microfilariae prevalence and its association with anemia among first-time blood donors in Lambaréné, Gabon. Balkan Med J. 2024;41:139–143. doi:10.4274/balkanmedj.galenos.2023.2023-9-86.

**143.** Paulo R, Brito M, Van-Dunem P, Martins A, Novak RJ, Jacob B, et al. Clinical, serological and DNA testing in Bengo Province, Angola further reveals low filarial endemicity and opportunities for disease elimination. Parasite Epidemiol Control. 2020;11:e00183. doi:10.1016/j.parepi.2020.e00183.

144. Pion SD, Montavon C, Chesnais CB, et al. Positivity of antigen tests used for diagnosis of lymphatic filariasis in individuals without *Wuchereria bancrofti* infection but with high *Loa loa* microfilaremia. *Am J Trop Med Hyg.* 2016;95(6):1417–1423. doi:10.4269/ajtmh.16-0547.

145. Pion SDS, Chesnais CB, Weil GJ, Fischer PU, Missamou F, Boussinesq M. Effect of 3 years of biannual mass drug administration with albendazole on lymphatic filariasis and soil-transmitted helminth infections: a community-based study in Republic of the Congo. *Lancet Infect Dis.* 2017;17:763–769. doi:10.1016/S1473-3099(17)30175-5.

146. Pion SDS, Chesnais CB, Awaca-Uvon NP, Vlaminck J, Abdou A, Kunyu-Shako B, et al. The impact of four years of semiannual treatments with albendazole alone on lymphatic filariasis and soil-transmitted helminth infections: a community-based study in the Democratic Republic of the Congo. *PLoS Negl Trop Dis.* 2020;14:e0008322. doi:10.1371/journal.pntd.0008322.

147. Rakers LJ, Emukah E, Kahansim B, Nwoke BEB, Miri ES, Griswold E, et al. Assessing hypoendemic onchocerciasis in *Loa loa* endemic areas of Southeast Nigeria. *Am J Trop Med Hyg.* 2020;103:2328–2335. doi:10.4269/ajtmh.20-0368.

148. Rebollo MP, Sambou SM, Thomas B, Biritwum N-K, Jaye MC, Kelly-Hope L, et al. Elimination of lymphatic filariasis in the Gambia. *PLoS Negl Trop Dis.* 2015;9:e0003642. doi:10.1371/journal.pntd.0003642.

149. Ruberanziza E, Mupfasoni D, Karibushi B, Rujeni N, Kabanda G, Kabera M, et al. Mapping of lymphatic filariasis in Rwanda. *J Lymphoedema.* 2009;4:20–23.

150. Sam-Wobo SO, Adeleke MA, Jayeola OA, Adeyi AO, Oluwole AS, Ikenga M, et al. Epidemiological evaluation of onchocerciasis along Ogun River System, southwest Nigeria. *J Vector Borne Dis.* 2012;49:101–104.

151. Sandri TL, Kreidenweiss A, Cavallo S, Weber D, Juhas S, Rodi M, et al. Molecular epidemiology of *Mansonella* species in Gabon. *J Infect Dis.* 2020;223:287–296. doi:10.1093/infdis/jiaa670.

152. Sangare MB, Coulibaly YI, Coulibaly SY, Coulibaly ME, Traore B, Dicko I, et al. A cross-sectional study of the filarial and *Leishmania* co-endemicity in two ecologically distinct settings in Mali. *Parasit Vectors.* 2018;11:18. doi:10.1186/s13071-017-2531-8.

153. Senkwe MN, Berta KK, Logora SMY, Sube J, Bidali A, Abe A, et al. Prevalence and factors associated with transmission of lymphatic filariasis in South Sudan: a cross-sectional quantitative study. *Pan Afr Med J.* 2022;42:9. doi:10.11604/pamj.supp.2022.42.1.33895.

154. Shawa ST, Mwase ET, Pedersen EM, Simonsen PE. Lymphatic filariasis in Luangwa District, South-East Zambia. *Parasit Vectors.* 2013;6:299. doi:10.1186/1756-3305-6-299.

155. Siewe Fodjo JN, Tatah G, Tabah EN, Ngarka L, Nfor LN, Chokote SE, et al. Epidemiology of onchocerciasis-associated epilepsy in the Mbam and Sanaga river valleys of Cameroon: impact of more than 13 years of ivermectin. *Infect Dis Poverty.* 2018;7:114. doi:10.1186/s40249-018-0497-1.

156. Siewe Fodjo JN, Mandro M, Mukendi D, Tepage F, Menon S, Nakato S, et al. Onchocerciasis-associated epilepsy in the Democratic Republic of Congo: Clinical description and relationship with microfilarial density. *PLoS Negl Trop Dis.* 2019;13:e0007300. doi:10.1371/journal.pntd.0007300.

157. Siewe JNF, Ukaga CN, Nwazor EO, Nwoke MO, Nwokeji MC, Onuoha BC, et al. Low prevalence of epilepsy and onchocerciasis after more than 20 years of ivermectin treatment in the Imo River Basin in Nigeria. *Infect Dis Poverty.* 2019;8:8. doi:10.1186/s40249-019-0517-9.

158. Siewe Fodjo JN, Njamnshi WY, Ngarka L, Nfor LN, Ayuk C, Mundih NN, et al. Association between Ov16 seropositivity and neurocognitive performance among children in rural Cameroon: a pilot study. *J Pediatr Neuropsychol.* 2021;7:192–202. doi:10.1007/s40817-021-00111-z.

159. Siewe Fodjo JN, Ngarka L, Njamnshi WY, Enyong PA, Zoung-Kanyi Bissek A-C, Njamnshi AK. Onchocerciasis in the Ntui Health District of Cameroon: epidemiological, entomological and parasitological findings in relation to elimination prospects. *Parasit Vectors.* 2022;15:444. doi:10.1186/s13071-022-05585-0.

160. Sumo L, Ntonifor NH, Afor AR, Bopda J, Bamou Heumou R, Ondoua Nganjou GS, et al. Loiasis is endemic in the Ndikinimeki Health District (Centre Region, Cameroon) but does not represent a hindrance to onchocerciasis elimination. *Acta Trop.* 2022;225:106218. doi:10.1016/j.actatropica.2021.106218.

161. Surakat OA, Babalola AS, Adeleke MA, Adeogun AO, Idowu OA, Sam-Wobo SO. Geospatial distribution and predictive modeling of onchocerciasis in Ogun State, Nigeria. *PLoS One.* 2023;18:e0281624. doi:10.1371/journal.pone.0281624.

162. Ta T-H, Moya L, Nguema J, Aparicio P, Miguel-Oteo M, Cenzual G, et al. Geographical distribution and species identification of human filariasis and onchocerciasis in Bioko Island, Equatorial Guinea. *Acta Trop.* 2018;180:12–17. doi:10.1016/j.actatropica.2017.12.030.

163. Tafatatha T, Taegtmeyer M, Ngwira B, Phiri A, Kondowe M, Piston W, et al. Human immunodeficiency virus, antiretroviral therapy and markers of lymphatic filariasis infection: a cross-sectional study in rural Northern Malawi. *PLoS Negl Trop Dis.* 2015;9:e0003825. doi:10.1371/journal.pntd.0003825.

164. Tekle AH, Elhassan E, Isiyaku S, Amazigo UV, Bush S, Noma M, et al. Impact of long-term treatment of onchocerciasis with ivermectin in Kaduna State, Nigeria: first evidence of the potential for elimination in the operational area of the African Programme for Onchocerciasis Control. *Parasit Vectors.* 2012;5:28. doi:10.1186/1756-3305-5-28.

165. Trevisan C, Hadermann A, Jada SR, Abong RA, Bol YY, Fodjo JNS, et al. Pregnant women, a potential reservoir for Onchocerca volvulus? A cross sectional study in Maridi County, South Sudan. *J Epidemiol Glob Health.* 2025;15:14. doi:10.1007/s44197-025-00357-2.

166. Tsapi EM, Todjom FG, Gamago G-A, Pone JW, Teukeng FFD. Prevalence of onchocerciasis after seven years of continuous community-directed treatment with ivermectin in the Ntui health district, Centre region, Cameroon. *Pan Afr Med J.* 2020;36:180. doi:10.11604/pamj.2020.36.180.20765.

167. Uttah EC. Prevalence of endemic bancroftian filariasis in the high altitude region of south-eastern Nigeria. *J Vector Borne Dis.* 2011;48:78–84.

168. Uttah E, Ibeh DC. Multiple filarial species microfilaraemia: a comparative study of areas with endemic and sporadic onchocerciasis. *J Vector Borne Dis.* 2011;48(4):197–204.

169. Van-Dúnem PJD, Brito M, Lemos M, Figueiredo JC. Socio-environmental factors associated with knowledge, attitude and practice and determinants of co-endemicity of filariasis in Chicala, Kuito province of Bié, Angola. 2023. doi:10.22533/AT.ED.1593982328119.

170. Van-Dúnem PJD, Brito M, Lemos M, Catumbela S, Pinda S, Capusso A, et al. Microscopic and biomolecular result of survey filarias diagnostic in the community of Angola in Chicala province of Bié. 2024. doi:10.22533/at.ed.813452413065.

171. Veletzky L, Eberhardt KA, Hergeth J, Stelzl DR, Zoleko Manego R, Mombo-Ngoma G, et al. Distinct loiasis infection states and associated clinical and hematological manifestations in patients from Gabon. *PLoS Negl Trop Dis.* 2022;16:e0010793. doi:10.1371/journal.pntd.0010793.

172. Wanji S, Akotshi DO, Mutro MN, Tepage F, Ukety TO, Diggle PJ, et al. Validation of the rapid assessment procedure for loiasis (RAPLOA) in the Democratic Republic of Congo. *Parasit Vectors.* 2012;5:25. doi:10.1186/1756-3305-5-25.

173. Wanji S, Amvongo-Adjia N, Koudou B, Njouendou AJ, et al. Cross-reactivity of filariasis ICT cards in areas of contrasting endemicity of *Loa loa* and *Mansonella perstans* in Cameroon: implications for shrinking of the lymphatic filariasis map in the Central African region. *PLoS Negl Trop Dis*. 2015;9(11):e0004184. doi:10.1371/journal.pntd.0004184. PMID:26544042; PMCID:PMC4636288.

174. Wanji S, Kengne-Ouafo JA, Esum ME, Chounna PWN, Tendongfor N, Adzemye BF, et al. Situation analysis of parasitological and entomological indices of onchocerciasis transmission in three drainage basins of the rain forest of South West Cameroon after a decade of ivermectin treatment. *Parasit Vectors.* 2015;8:202. doi:10.1186/s13071-015-0817-2.175. Wanji S, Kengne-Ouafo JA, Esum ME, Chounna PWN, Adzemye BF, Eyong JEE, et al. Relationship between oral declaration on adherence to ivermectin treatment and parasitological indicators of onchocerciasis in an area of persistent transmission despite a decade of mass drug administration in Cameroon. *Parasit Vectors.* 2015;8:667. doi:10.1186/s13071-015-1283-6.

176. Wanji S, Tayong DB, Layland LE, Datchoua Poutcheu FR, Ndongmo WPC, Kengne-Ouafo JA, et al. Update on the distribution of Mansonella perstans in the southern part of Cameroon: influence of ecological factors and mass drug administration with ivermectin. *Parasit Vectors.* 2016;9:311. doi:10.1186/s13071-016-1595-1.

177. Wilson NO, Badara Ly A, Cama VA, Cantey PT, Cohn D, Diawara L, et al. Evaluation of lymphatic filariasis and onchocerciasis in three Senegalese Districts treated for onchocerciasis with ivermectin. *PLoS Negl Trop Dis.* 2016;10:e0005198. doi:10.1371/journal.pntd.0005198.

178. Won KY, Sambou S, Barry A, Robinson K, Jaye M, Sanneh B, Sanyang A, Gass K, Lammie PJ, Rebollo M. Use of antibody tools to provide serologic evidence of elimination of lymphatic filariasis in the Gambia. *Am J Trop Med Hyg.* 2018;98(1):15–20. doi:10.4269/ajtmh.17-0371.

179. Yaya G, Kobangué L, Kémata B, Gallé D, Grésenguet G. Élimination ou contrôle de l’onchocercose en Afrique? Cas du village de Gami en République centrafricaine. *Bull Soc Pathol Exot.* 2014;107:188–193. doi:10.1007/s13149-014-0363-8.

180. Yokoly FN, Zahouli JBZ, Méite A, Opoku M, Kouassi BL, de Souza DK, et al. Low transmission of *Wuchereria bancrofti* in cross-border districts of Côte d’Ivoire: a great step towards lymphatic filariasis elimination in West Africa. *PLoS One.* 2020;15:e0231541. doi:10.1371/journal.pone.0231541.
