# Supplementary material for: Human Filariasis in Africa (2000–2025): Changing epidemiology, uneven diagnostic progress, and persistent neglect
Source: PLoS Negl Trop Dis. 2026 Apr 7;20(4):e0014200. doi: 10.1371/journal.pntd.0014200 (PMC13082706; doi:10.1371/journal.pntd.0014200)
Supplement: S4 Appendix — (DOCX) [file pntd.0014200.s004.docx]

**S4 Appendix . Additional statistical calculations and forest plots.**

**Table A.** Pooled prevalence of filarial species by five-year intervals, with 95% confidence intervals and heterogeneity measures (I², tau²).

| **Species** | **Period** | **Pooled_prev** | **CI_lower** | **CI_upper** | **I^2^** | **tau^2^** |
| --- | --- | --- | --- | --- | --- | --- |
| Onchocerciasis | 2000-2004 | 0.016108 | 0.000133 | 0.668278 | 91.65024 | 11.12551 |
| Onchocerciasis | 2005-2009 | 0.04394 | 0.0217 | 0.086948 | 98.46206 | 1.221483 |
| Onchocerciasis | 2010-2014 | 0.031904 | 0.015849 | 0.063179 | 99.72073 | 2.320373 |
| Onchocerciasis | 2015-2019 | 0.062886 | 0.037754 | 0.102956 | 99.68082 | 1.481418 |
| Onchocerciasis | 2020-2025 | 0.069128 | 0.027555 | 0.162915 | 99.08499 | 2.807423 |
| Loiasis | 2000-2004 | 0.105708 | 0.081029 | 0.136786 | 0 | 0 |
| Loiasis | 2005-2009 | 0.252661 | 0.213582 | 0.296197 | 88.60333 | 0.03148 |
| Loiasis | 2010-2014 | 0.058209 | 0.035491 | 0.094049 | 98.92596 | 0.645134 |
| Loiasis | 2015-2019 | 0.126985 | 0.065241 | 0.232621 | 99.87117 | 1.939995 |
| Loiasis | 2020-2025 | 0.052977 | 0.023471 | 0.115198 | 98.32504 | 1.158353 |
| Lymphatic filariasis | 2000-2004 | 0.10572 | 0.06595 | 0.16523 | 97.35111 | 0.197688 |
| Lymphatic filariasis | 2005-2009 | 0.026629 | 0.01023 | 0.067524 | 99.32273 | 1.464094 |
| Lymphatic filariasis | 2010-2014 | 0.011982 | 0.006301 | 0.022666 | 98.77775 | 2.138471 |
| Lymphatic filariasis | 2015-2019 | 0.016111 | 0.005372 | 0.047299 | 97.49383 | 3.473876 |
| Lymphatic filariasis | 2020-2025 | 0.004673 | 0.001508 | 0.014385 | 0 | 0 |
| Mansonellosis | 2000-2004 | 0.048864 | 0.021636 | 0.106626 | 69.5238 | 0.258954 |
| Mansonellosis | 2005-2009 | 0.152954 | 0.072023 | 0.295836 | 99.56736 | 0.553694 |
| Mansonellosis | 2010-2014 | 0.118724 | 0.060924 | 0.218596 | 99.61278 | 1.673197 |
| Mansonellosis | 2015-2019 | 0.08686 | 0.038412 | 0.184678 | 99.31074 | 1.514431 |
| Mansonellosis | 2020-2025 | 0.024754 | 7.97E-05 | 0.889908 | 98.04236 | 16.96209 |

Pooled prevalence estimates for key filarial species by five-year periods. Values represent the pooled prevalence (proportion of positive cases) with 95% confidence intervals (CI) and measures of heterogeneity (I², tau²). Estimates were calculated using random-effects meta-analysis. Extreme values of tau² in some periods reflect high between-study variability due to small numbers of studies or sample sizes. This table complements the forest plots (Supplementary Figure 1-4 S4.).


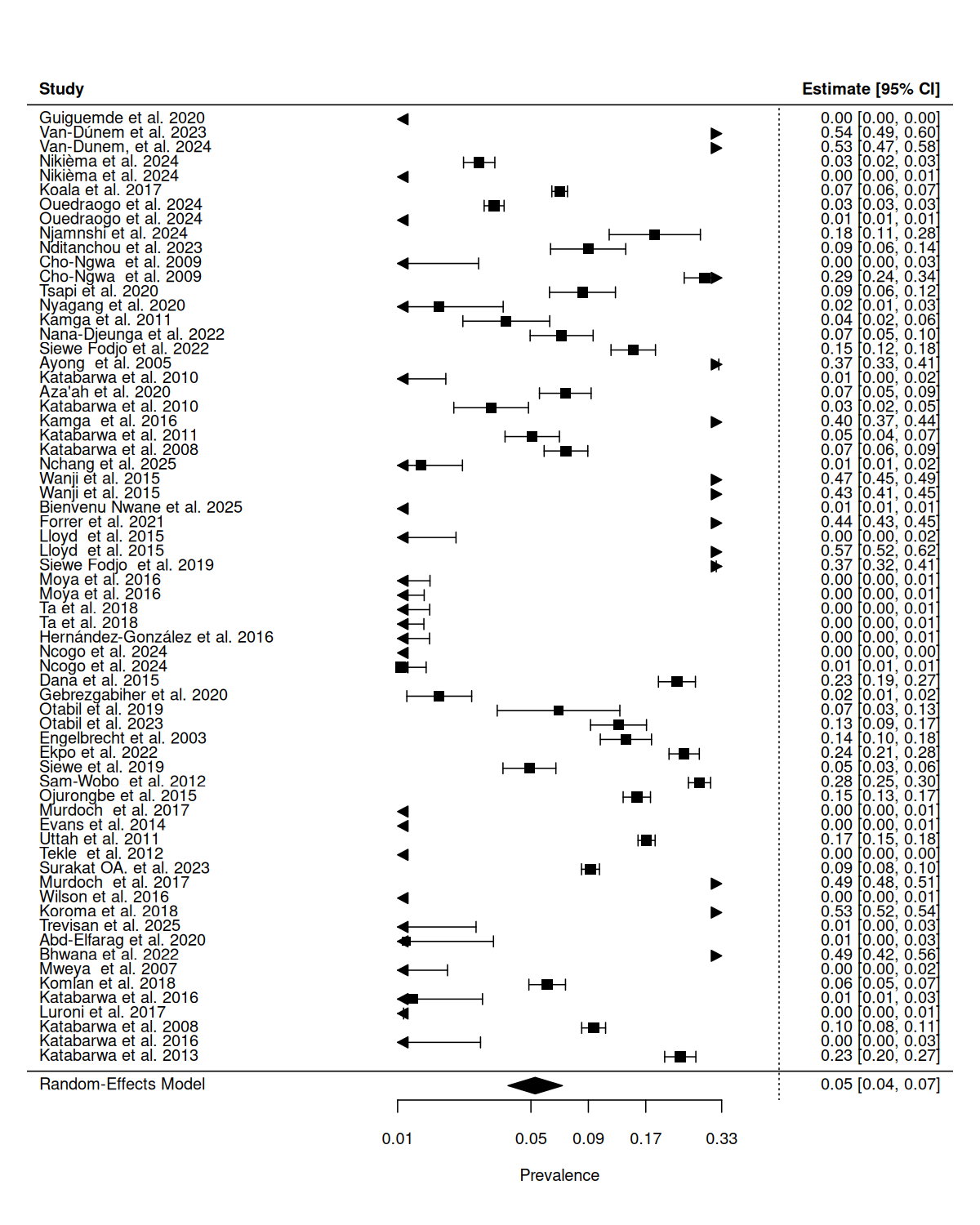


**Fig. A.** Forest plot of pooled prevalence for onchocerciasis with 95% confidence intervals


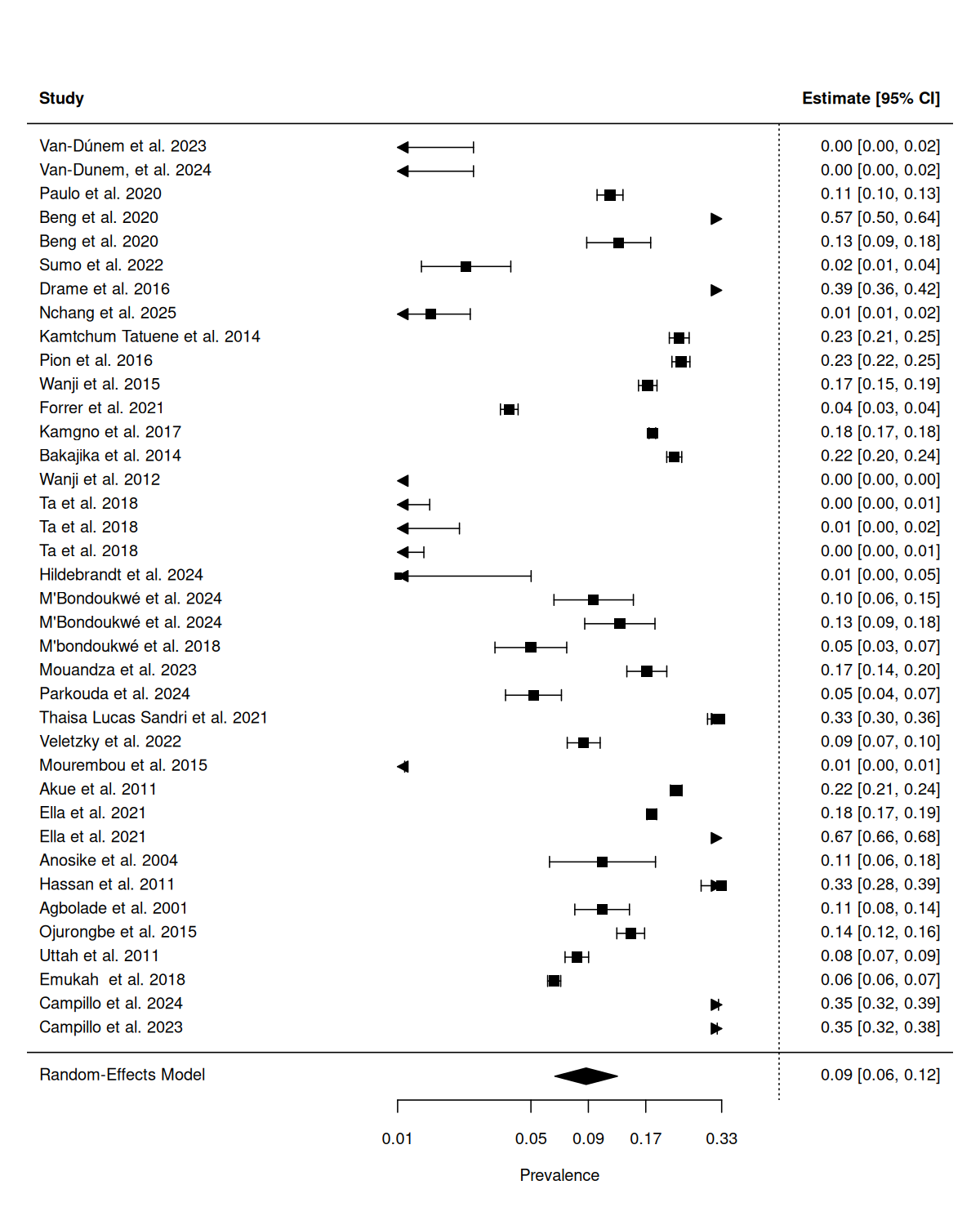


**Fig. B.** Forest plot of pooled prevalence for loiasis with 95% confidence intervals


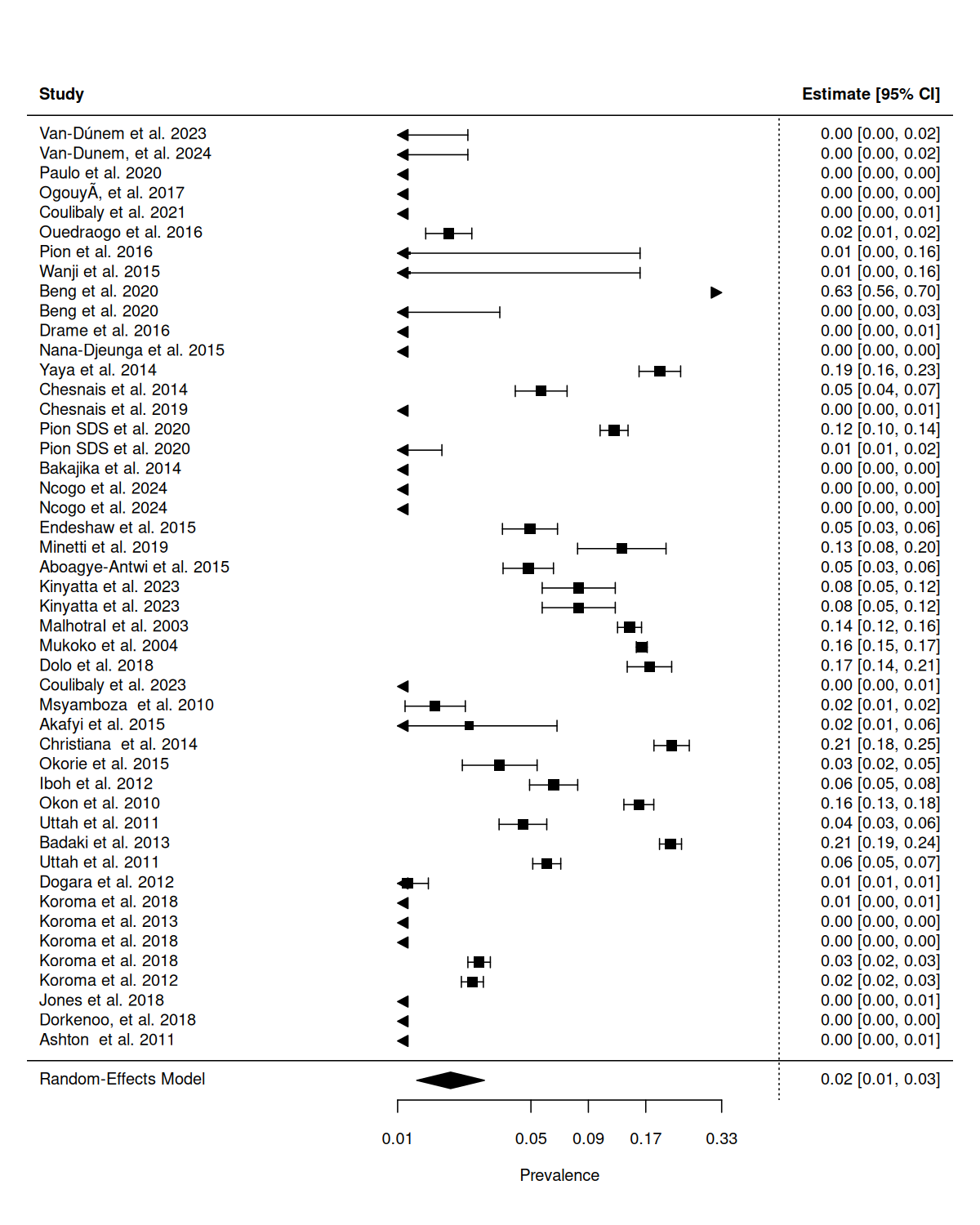


**Fig. C.** Forest plot of pooled prevalence for lymphatic filariasis with 95% confidence intervals


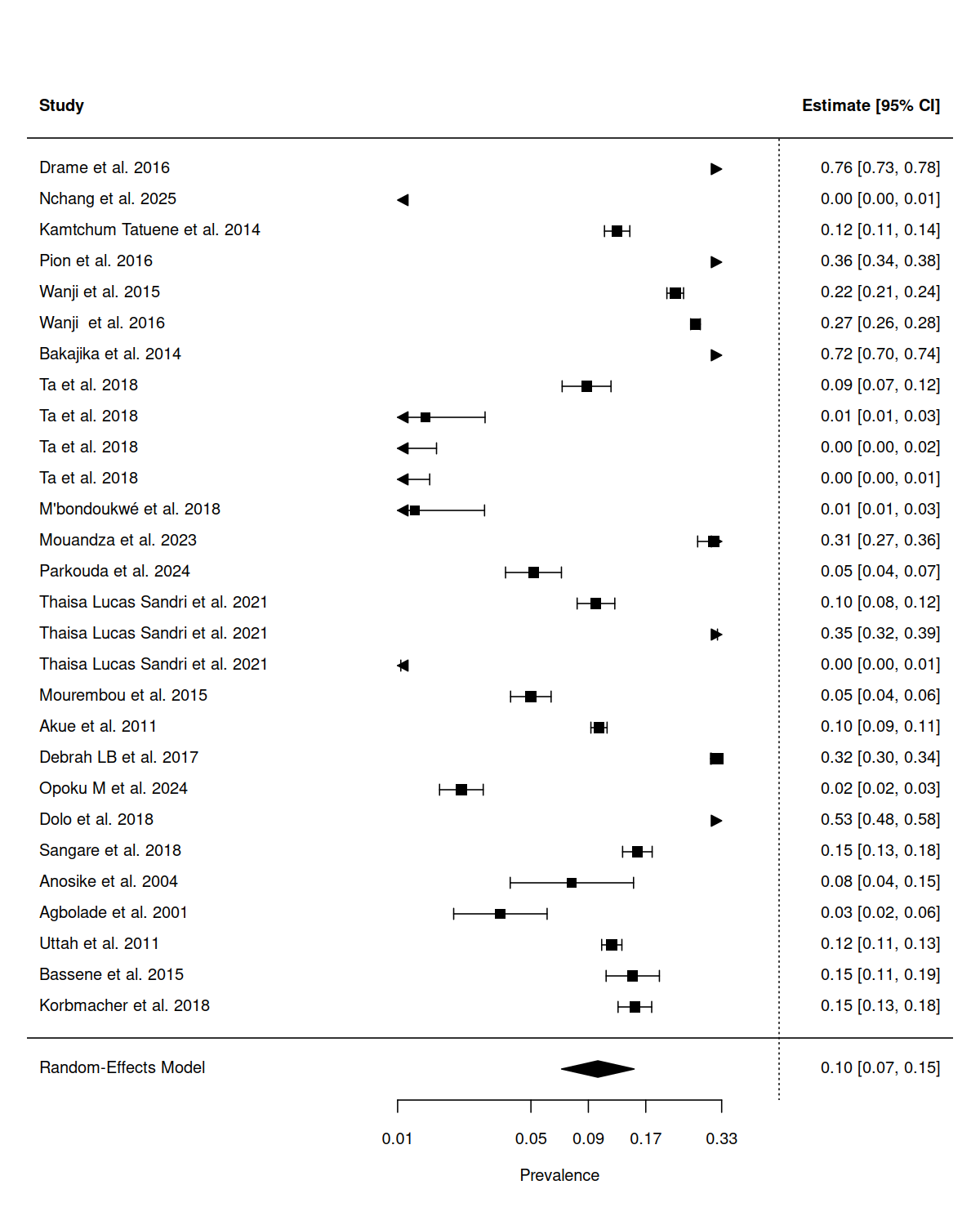


**Fig. D.** Forest plot of pooled prevalence for mansonellosis with 95% confidence intervals

**Table B.** Meta-regression of diagnostic method (microscopy vs. molecular) on prevalence estimates for active infections across filarial species. Serological results are excluded.

| \|  \| \| --- \|  \| **Species** \| \| --- \| | **Coefficient** | \|  \| \| --- \|  \| **Logit Estimate** \| \| --- \| | **Standard Error (SE)** | \|  \| \| --- \|  \| **z-value** \| \| --- \| | \|  \| \| --- \|  \| **p-value** \| \| --- \| | \|  \| \| --- \|  \| **95% CI Lower** \| \| --- \| | \|  \| \| --- \|  \| **95% CI Upper** \| \| --- \| |
| --- | --- | --- | --- | --- | --- | --- | --- | --- | --- | --- | --- | --- | --- | --- | --- | --- | --- | --- | --- |
| Onchocerciasis | intrcpt | -3.14112 | 0.296419 | -10.5969 | 3.08E-26 | -3.72209 | -2.56014 |
| Onchocerciasis | factor(diagnostic_method)molecular | -0.90014 | 1.222112 | -0.73654 | 0.461401 | -3.29543 | 1.495159 |
| Loiasis | intrcpt | -2.44647 | 0.338961 | -7.21755 | 5.29E-13 | -3.11082 | -1.78211 |
| Loiasis | factor(diagnostic_method)molecular | -0.17639 | 0.78972 | -0.22336 | 0.823253 | -1.72422 | 1.371428 |
| Lymphtaic filariasis | intrcpt | -4.28695 | 0.375871 | -11.4054 | 3.93E-30 | -5.02364 | -3.55025 |
| Lymphtaic filariasis | factor(diagnostic_method)molecular | -0.03597 | 1.086163 | -0.03311 | 0.973584 | -2.16481 | 2.092875 |
| Mansonellosis | intrcpt | -2.39641 | 0.466154 | -5.14081 | 2.74E-07 | -3.31005 | -1.48276 |
| Mansonellosis. | factor(diagnostic_method)molecular | 0.172125 | 0.815949 | 0.210951 | 0.832925 | -1.42711 | 1.771356 |

**Table C.** Prevalence of lymphatic filariasis, onchocerciasis, loiasis, and mansonellosis by country, period, and ESPEN-reported endemicity and MDA coverage (WHO AFRO ESPEN Data Portal).

| **country** | **species** | **period5** | **n_studies** | **total_examined** | **total_positive** | **prevalence**  **[%]** | **ESPEN-endemic** | **ESPEN-MDA delivery** |
| --- | --- | --- | --- | --- | --- | --- | --- | --- |
| Angola | onchocerciasis | 2020-2025 | 2 | 640 | 343 | 53.59 | Endemic | MDA delivered but not 100% geographical coverage |
| Burkina Faso | onchocerciasis | 2020-2025 | 2 | 4498 | 5 | 0.11 | Endemic | MDA 100% geographical coverage |
| Cameroon | onchocerciasis | 2020-2025 | 8 | 7524 | 242 | 3.22 | Endemic | MDA delivered but not 100% geographical coverage |
| Central African Republic | onchocerciasis | 2020-2025 | 1 | 259 | 23 | 8.88 | Endemic | MDA delivered but not 100% geographical coverage |
| Gabon | onchocerciasis | 2020-2025 | 1 | 359 | 87 | 24.23 | Endemic | MDA not delivered |
| Ghana | onchocerciasis | 2020-2025 | 2 | 570 | 102 | 17.89 | Endemic | MDA 100% geographical coverage |
| Nigeria | onchocerciasis | 2020-2025 | 2 | 13345 | 164 | 1.23 | Endemic | MDA delivered but not 100% geographical coverage |
| Republic of Congo | onchocerciasis | 2020-2025 | 1 | 971 | 22 | 2.27 | Endemic | MDA 100% geographical coverage |
| South Sudan | onchocerciasis | 2020-2025 | 4 | 1058 | 112 | 10.59 | Endemic | MDA delivered but not 100% geographical coverage |
| Tanzania | onchocerciasis | 2020-2025 | 2 | 32495 | 4205 | 12.94 | Endemic | MDA not delivered |
| Burkina Faso | onchocerciasis | 2015-2019 | 2 | 17528 | 105 | 0.60 | Endemic | MDA 100% geographical coverage |
| Cameroon | onchocerciasis | 2015-2019 | 10 | 30653 | 9642 | 31.46 | Endemic | MDA delivered but not 100% geographical coverage |
| Democratic Republic of the Congo | onchocerciasis | 2015-2019 | 3 | 1666 | 548 | 32.89 | Endemic | MDA 100% geographical coverage |
| Equatorial Guinea | onchocerciasis | 2015-2019 | 3 | 11853 | 52 | 0.44 | Endemic | MDA not delivered |
| Ethiopia | onchocerciasis | 2015-2019 | 2 | 4944 | 66 | 1.33 | Endemic | MDA delivered but not 100% geographical coverage |
| Gabon | onchocerciasis | 2015-2019 | 1 | 7751 | 1046 | 13.50 | Endemic | MDA not delivered |
| Ghana | onchocerciasis | 2015-2019 | 1 | 114 | 7 | 6.14 | Endemic | MDA delivered but not 100% geographical coverage |
| Malawi | onchocerciasis | 2015-2019 | 2 | 12025 | 55 | 0.46 | Endemic | MDA 100% geographical coverage |
| Nigeria | onchocerciasis | 2015-2019 | 8 | 36768 | 4538 | 12.34 | Endemic | MDA delivered but not 100% geographical coverage |
| Sierra Leone | onchocerciasis | 2015-2019 | 2 | 20257 | 362 | 1.79 | Endemic | MDA 100% geographical coverage |
| South Sudan | onchocerciasis | 2015-2019 | 2 | 462 | 38 | 8.23 | Endemic | MDA delivered but not 100% geographical coverage |
| Tanzania | onchocerciasis | 2015-2019 | 1 | 210 | 103 | 49.05 | Endemic | MDA 100% geographical coverage |
| Togo | onchocerciasis | 2015-2019 | 3 | 4365 | 1303 | 29.85 | Endemic | MDA 100% geographical coverage |
| Angola | onchocerciasis | 2010-2014 | 1 | 1567 | 74 | 4.72 | Endemic | MDA not delivered |
| Burkina Faso | onchocerciasis | 2010-2014 | 3 | 20043 | 845 | 4.22 | Endemic | MDA 100% geographical coverage |
| Cameroon | onchocerciasis | 2010-2014 | 4 | 4412 | 1244 | 28.20 | Endemic | MDA delivered but not 100% geographical coverage |
| Côte d'Ivoire | onchocerciasis | 2010-2014 | 2 | 738 | 211 | 28.59 | Endemic | MDA delivered but not 100% geographical coverage |
| Equatorial Guinea | onchocerciasis | 2010-2014 | 7 | 3784 | 87 | 2.30 | Endemic | MDA not delivered |
| Ethiopia | onchocerciasis | 2010-2014 | 1 | 440 | 99 | 22.50 | Endemic | MDA delivered but not 100% geographical coverage |
| Nigeria | onchocerciasis | 2010-2014 | 1 | 2579 | 433 | 16.79 | Endemic | MDA delivered but not 100% geographical coverage |
| Senegal | onchocerciasis | 2010-2014 | 2 | 2123 | 79 | 3.72 | Endemic | MDA 100% geographical coverage |
| Sierra Leone | onchocerciasis | 2010-2014 | 1 | 7116 | 3779 | 53.11 | Endemic | MDA 100% geographical coverage |
| Togo | onchocerciasis | 2010-2014 | 2 | 2929 | 940 | 32.09 | Endemic | MDA 100% geographical coverage |
| Uganda | onchocerciasis | 2010-2014 | 4 | 44166 | 564 | 1.27 | Endemic | MDA 100% geographical coverage |
| Sudan | onchocerciasis | 2015-2019 | 1 | 2848 | 0 | 0.00 | N/A | MDA not delivered |
| Cameroon | onchocerciasis | 2005-2009 | 8 | 3884 | 1039 | 26.75 | N/A | N/A |
| Nigeria | onchocerciasis | 2005-2009 | 4 | 9160 | 378 | 4.13 | N/A | N/A |
| Uganda | onchocerciasis | 2005-2009 | 3 | 5916 | 222 | 3.75 | N/A | N/A |
| Uganda | onchocerciasis | 2005-2009 | 1 | 294 | 0 | 0.00 | N/A | N/A |
| Kenya | onchocerciasis | 2000-2004 | 1 | 1522 | 569 | 37.39 | N/A | N/A |
| Nigeria | onchocerciasis | 2000-2004 | 1 | 341 | 46 | 13.49 | N/A | N/A |
| Tanzania | onchocerciasis | 2000-2004 | 1 | 438 | 0 | 0.00 | N/A | N/A |
| Cameroon | mansonellosis | 2020-2025 | 1 | 1184 | 1 | 0.08 | Not monitored | |
| Gabon | mansonellosis | 2020-2025 | 1 | 471 | 147 | 31.21 | Not monitored | |
| Cameroon | mansonellosis | 2015-2019 | 1 | 2190 | 486 | 22.19 | Not monitored | |
| Gabon | mansonellosis | 2015-2019 | 5 | 4643 | 481 | 10.36 | Not monitored | |
| Ghana | mansonellosis | 2015-2019 | 1 | 2672 | 56 | 2.10 | Not monitored | |
| Malawi | mansonellosis | 2015-2019 | 1 | 372 | 197 | 52.96 | Not monitored | |
| Cameroon | mansonellosis | 2010-2014 | 2 | 2897 | 1478 | 51.02 | Not monitored | |
| Democratic Republic of the Congo | mansonellosis | 2010-2014 | 1 | 2724 | 1961 | 71.99 | Not monitored | |
| Equatorial Guinea | mansonellosis | 2010-2014 | 4 | 2172 | 57 | 2.62 | Not monitored | |
| Gabon | mansonellosis | 2010-2014 | 1 | 451 | 5 | 1.11 | Not monitored | |
| Ghana | mansonellosis | 2010-2014 | 1 | 2247 | 726 | 32.31 | Not monitored | |
| Malawi | mansonellosis | 2010-2014 | 1 | 930 | 142 | 15.27 | Not monitored | |
| Nigeria | mansonellosis | 2010-2014 | 1 | 2496 | 292 | 11.70 | Not monitored | |
| Senegal | mansonellosis | 2010-2014 | 1 | 297 | 43 | 14.48 | Not monitored | |
| Togo | mansonellosis | 2010-2014 | 1 | 733 | 109 | 14.87 | Not monitored | |
| Cameroon | mansonellosis | 2005-2009 | 2 | 7232 | 1715 | 23.71 | Not monitored | |
| Gabon | mansonellosis | 2005-2009 | 1 | 4392 | 448 | 10.20 | Not monitored | |
| Nigeria | mansonellosis | 2000-2004 | 2 | 471 | 19 | 4.03 | Not monitored | |
| Togo | lymphatic filariasis | 2015-2019 | 3 | 15348 | 137 | 0.89 | Eliminated | Post validation surveillance |
| Angola | lymphatic filariasis | 2020-2025 | 2 | 640 | 2 | 0.31 | Endemic | MDA delivered but not 100% geographical coverage |
| Central African Republic | lymphatic filariasis | 2020-2025 | 1 | 259 | 5 | 1.93 | Endemic | MDA delivered but not 100% geographical coverage |
| Tanzania | lymphatic filariasis | 2020-2025 | 1 | 590 | 30 | 5.08 | Endemic | MDA not delivered |
| Zimbabwe | lymphatic filariasis | 2020-2025 | 1 | 18488 | 74 | 0.40 | Endemic | MDA 100% geographical coverage |
| Benin | lymphatic filariasis | 2015-2019 | 2 | 12402 | 46 | 0.37 | Endemic | MDA 100% geographical coverage |
| Burkina Faso | lymphatic filariasis | 2015-2019 | 1 | 1649 | 3 | 0.18 | Endemic | MDA 100% geographical coverage |
| Cameroon | lymphatic filariasis | 2015-2019 | 4 | 14904 | 346 | 2.32 | Endemic | MDA 100% geographical coverage |
| Côte d'Ivoire | lymphatic filariasis | 2015-2019 | 1 | 2409 | 14 | 0.58 | Endemic | MDA 100% geographical coverage |
| Democratic Republic of the Congo | lymphatic filariasis | 2015-2019 | 2 | 2580 | 122 | 4.73 | Endemic | MDA delivered but not 100% geographical coverage |
| Equatorial Guinea | lymphatic filariasis | 2015-2019 | 3 | 11853 | 182 | 1.54 | Endemic | MDA not delivered |
| Ethiopia | lymphatic filariasis | 2015-2019 | 1 | 3377 | 7 | 0.21 | Endemic | MDA delivered but not 100% geographical coverage |
| Gabon | lymphatic filariasis | 2015-2019 | 1 | 7751 | 78 | 1.01 | Endemic | MDA not delivered |
| Ghana | lymphatic filariasis | 2015-2019 | 4 | 9466 | 1626 | 17.18 | Endemic | MDA 100% geographical coverage |
| Kenya | lymphatic filariasis | 2015-2019 | 4 | 1048 | 230 | 21.95 | Endemic | MDA 100% geographical coverage |
| Madagascar | lymphatic filariasis | 2015-2019 | 1 | 3562 | 11 | 0.31 | Endemic | MDA delivered but not 100% geographical coverage |
| Nigeria | lymphatic filariasis | 2015-2019 | 5 | 13394 | 276 | 2.06 | Endemic | MDA delivered but not 100% geographical coverage |
| Sierra Leone | lymphatic filariasis | 2015-2019 | 1 | 7105 | 7 | 0.10 | Endemic | MDA delivered but not 100% geographical coverage |
| South Sudan | lymphatic filariasis | 2015-2019 | 1 | 9213 | 147 | 1.60 | Endemic | MDA delivered but not 100% geographical coverage |
| Tanzania | lymphatic filariasis | 2015-2019 | 4 | 3666 | 32 | 0.87 | Endemic | MDA delivered but not 100% geographical coverage |
| Uganda | lymphatic filariasis | 2015-2019 | 1 | 101 | 1 | 0.99 | Endemic | MDA 100% geographical coverage |
| Angola | lymphatic filariasis | 2010-2014 | 2 | 3232 | 0 | 0.00 | Endemic | MDA not delivered |
| Burkina Faso | lymphatic filariasis | 2010-2014 | 2 | 4758 | 58 | 1.22 | Endemic | MDA 100% geographical coverage |
| Cameroon | lymphatic filariasis | 2010-2014 | 4 | 8610 | 45 | 0.52 | Endemic | MDA 100% geographical coverage |
| Central African Republic | lymphatic filariasis | 2010-2014 | 1 | 393 | 75 | 19.08 | Endemic | MDA not delivered |
| Congo | lymphatic filariasis | 2010-2014 | 4 | 2982 | 340 | 11.40 | Endemic | MDA delivered but not 100% geographical coverage |
| Democratic Republic of the Congo | lymphatic filariasis | 2010-2014 | 6 | 9644 | 980 | 10.16 | Endemic | MDA delivered but not 100% geographical coverage |
| Ethiopia | lymphatic filariasis | 2010-2014 | 1 | 774 | 36 | 4.65 | Endemic | MDA delivered but not 100% geographical coverage |
| Guinea | lymphatic filariasis | 2010-2014 | 1 | 611 | 0 | 0.00 | Endemic | MDA delivered but not 100% geographical coverage |
| Nigeria | lymphatic filariasis | 2010-2014 | 8 | 12882 | 1054 | 8.18 | Endemic | MDA delivered but not 100% geographical coverage |
| Senegal | lymphatic filariasis | 2010-2014 | 2 | 2262 | 13 | 0.57 | Endemic | MDA 100% geographical coverage |
| Sierra Leone | lymphatic filariasis | 2010-2014 | 3 | 16276 | 61 | 0.37 | Endemic | MDA 100% geographical coverage |
| Tanzania | lymphatic filariasis | 2010-2014 | 7 | 8154 | 756 | 9.27 | Endemic | MDA delivered but not 100% geographical coverage |
| Uganda | lymphatic filariasis | 2010-2014 | 2 | 2608 | 52 | 1.99 | Endemic | MDA delivered but not 100% geographical coverage |
| Zambia | lymphatic filariasis | 2010-2014 | 4 | 18064 | 558 | 3.08 | Endemic | MDA delivered but not 100% geographical coverage |
| Sudan | lymphatic filariasis | 2015-2019 | 3 | 8544 | 1 | 0.01 | N/A | MDA not delivered |
| Egypt | lymphatic filariasis | 2010-2014 | 2 | 2642 | 26 | 0.98 | N/A | N/A |
| Gambia | lymphatic filariasis | 2015-2019 | 2 | 5224 | 78 | 1.49 | Non-endemic | Non-endemic |
| Gambia | lymphatic filariasis | 2010-2014 | 1 | 3180 | 0 | 0.00 | Non-endemic | Non-endemic |
| Togo | lymphatic filariasis | 2010-2014 | 4 | 46132 | 203 | 0.44 | Surveillance | Post-MDA surveillance |
| Cameroon | lymphatic filariasis | 2005-2009 | 2 | 37529 | 390 | 1.04 | N/A | N/A |
| Ghana | lymphatic filariasis | 2005-2009 | 1 | 625 | 12 | 1.92 | N/A | N/A |
| Kenya | lymphatic filariasis | 2005-2009 | 1 | 1079 | 10 | 0.93 | N/A | N/A |
| Malawi | lymphatic filariasis | 2005-2009 | 3 | 4762 | 307 | 6.45 | N/A | N/A |
| Nigeria | lymphatic filariasis | 2005-2009 | 5 | 39727 | 1453 | 3.66 | N/A | N/A |
| Rwanda | lymphatic filariasis | 2005-2009 | 1 | 797 | 1 | 0.13 | N/A | N/A |
| Sierra Leone | lymphatic filariasis | 2005-2009 | 3 | 19503 | 849 | 4.35 | N/A | N/A |
| Tanzania | lymphatic filariasis | 2005-2009 | 2 | 3078 | 714 | 23.20 | N/A | N/A |
| Ghana | lymphatic filariasis | 2000-2004 | 2 | 1802 | 118 | 6.55 | N/A | N/A |
| Kenya | lymphatic filariasis | 2000-2004 | 3 | 9461 | 1555 | 16.44 | N/A | N/A |
| Malawi | lymphatic filariasis | 2000-2004 | 1 | 1139 | 244 | 21.42 | N/A | N/A |
| Nigeria | lymphatic filariasis | 2000-2004 | 2 | 682 | 123 | 18.04 | N/A | N/A |
| Zambia | lymphatic filariasis | 2000-2004 | 2 | 13943 | 1195 | 8.57 | N/A | N/A |
| Ghana | lymphatic filariasis | 2000-2005 | 2 | 18983 | 39 | 0.21 | N/A | N/A |
| Angola | loiasis | 2020-2025 | 2 | 640 | 0 | 0.00 | Endemic | N/A |
| Cameroon | loiasis | 2020-2025 | 2 | 1787 | 30 | 1.68 | Endemic | N/A |
| Gabon | loiasis | 2020-2025 | 2 | 621 | 80 | 12.88 | Endemic | N/A |
| Republic of Congo | loiasis | 2020-2025 | 4 | 3903 | 2288 | 58.62 | Endemic | N/A |
| Cameroon | loiasis | 2015-2019 | 4 | 26130 | 3377 | 12.92 | Endemic | N/A |
| Gabon | loiasis | 2015-2019 | 8 | 20091 | 7044 | 35.06 | Endemic | N/A |
| Nigeria | loiasis | 2015-2019 | 2 | 11720 | 817 | 6.97 | Non-endemic | N/A |
| Angola | loiasis | 2010-2014 | 1 | 1543 | 177 | 11.47 | Endemic | N/A |
| Cameroon | loiasis | 2010-2014 | 3 | 5087 | 1216 | 23.90 | Endemic | N/A |
| Democratic Republic of the Congo | loiasis | 2010-2014 | 2 | 10824 | 607 | 5.61 | Endemic | N/A |
| Equatorial Guinea | loiasis | 2010-2014 | 3 | 1629 | 5 | 0.31 | Endemic | N/A |
| Gabon | loiasis | 2010-2014 | 1 | 451 | 21 | 4.66 | Endemic | N/A |
| Nigeria | loiasis | 2010-2014 | 1 | 2579 | 206 | 7.99 | Non-endemic | N/A |
| Cameroon | loiasis | 2005-2009 | 1 | 1511 | 348 | 23.03 | N/A | N/A |
| Gabon | loiasis | 2005-2009 | 1 | 4392 | 984 | 22.40 | N/A | N/A |
| Nigeria | loiasis | 2005-2009 | 1 | 286 | 95 | 33.22 | N/A | N/A |
| Nigeria | loiasis | 2000-2004 | 2 | 471 | 49 | 10.40 | N/A | N/A |

*Data source:* *World Health Organization Regional Office for Africa. ESPEN Data Portal: Documentation. Brazzaville: WHO Regional Office for Africa; 2022 (cited 2025 Feb 12). Available from:* [*https://espen.afro.who.int/*](https://espen.afro.who.int/)
