# Supplementary material for: Human Filariasis in Africa (2000–2025): Changing epidemiology, uneven diagnostic progress, and persistent neglect
Source: PLoS Negl Trop Dis. 2026 Apr 7;20(4):e0014200. doi: 10.1371/journal.pntd.0014200 (PMC13082706; doi:10.1371/journal.pntd.0014200)
Supplement: Attachment — Submitted filename: Review report.docx [file pntd.0014200.s006.docx]

**RE: PNTD-D-25-02073**

The authors present a systematic review assessing the epidemiological trends and diagnostic trend of human filariasis in Africa over a 25-year period. The topic is highly relevant to public health and NTD elimination agendas. The manuscript is generally well-structured, the PRISMA flow is clear, and the conclusions address important gaps in surveillance and equity. However, several critical issues require substantial revision before the manuscript can be considered for publication.

**Major comments**

1. Lack of Quantitative Synthesis: No pooled estimates, confidence intervals, or measures of heterogeneity (I²) are presented in the main text or figures, despite the use of ‘metaprop’.
2. Alternatively, authors should provide forest plots or summary tables of pooled prevalence estimates for key species/periods in supplementary materials (not as a raw data in S2).
3. Formally test for subgroup differences (e.g., microscopy vs. molecular) using meta-regression, even if limited to more homogeneous subsets of studies. This is crucial to support claims that diagnostic method influences prevalence estimates.
4. The figures 3-6 show prevalence trends but without measures of variance (e.g., 95% CIs) or accounting for differing numbers of studies per period. At least, error bar should be added.
5. The pie charts for diagnostic methods should be accompanied by absolute numbers (n) in the legend or on the charts themselves.
6. The maps included in figure 3-6 required distinctive colour contrast and also needed to include the keys to interpret the colours.
7. The authors reported temporal trends, diagnostic methods, and geographic distribution of studies of species. However, geographical distribution should be a reflection of prevalence of the species in each region or country. This could be the ideal map for figures 3-6, instead of ‘geographical distribution of studies’. It is surprising that the authors did not analyze the prevalence of each species according to country or at least, representatives of top 5 or 10 countries with high prevalence should have been presented.
8. The discussion lacks depth and centers on mere description. The discussion describes the problems (geographic bias, diagnostic limitations) but falls short of providing a novel or actionable synthesis.
9. No indication in the manuscript that the study protocol was registered in PROSPERO. This is not acceptable.

**Minor Comments**

1. No clear objective(s) of the study stated in both abstract & introduction.
2. The keywords are redundant. Human filariasis; Neglected tropical diseases (NTDs); Africa; Lymphatic filariasis; Microscopy; Molecular diagnostics.
3. PECO Framework: The "Comparator" is vague. Specify that the comparison is implicit: trends over time and across diagnostic methodologies.
4. Clarify what constitutes a "data point." Is it a unique pathogen-study-population combination? Ensure this is consistently applied.
5. The authors need to avoid assertive statements on MDAs, since the study did not compare or report MDAs coverage or status for each region.
6. Limitations: ‘Many regions of Africa, particularly Northern and parts of Southern Africa, are underrepresented in indexed databases due to limited research infrastructure, scarce funding, and reduced opportunities for local investigators to publish in high-impact journals.’ This is a vague statement without any evidence to support.
