# Supplementary material for: Human Filariasis in Africa (2000–2025): Changing epidemiology, uneven diagnostic progress, and persistent neglect
Source: PLoS Negl Trop Dis. 2026 Apr 7;20(4):e0014200. doi: 10.1371/journal.pntd.0014200 (PMC13082706; doi:10.1371/journal.pntd.0014200)
Supplement: Attachment — Submitted filename: Response to Reviewers.docx [file pntd.0014200.s008.docx]

Dear PLOS Editorial Office,

Thank you for flagging the potential copyright issues regarding the map figures (Figures 2–6). All maps were generated by the authors in R using openly available base layers from Natural Earth. The base layer used for country boundaries is: Natural Earth, “Admin 0 – Countries” (1:10m cultural vectors): <https://www.naturalearthdata.com/downloads/10m-cultural-vectors/10m-admin-0-countries/>. The terms of use / license information is available here: https://www.naturalearthdata.com/about/terms-of-use/ (public domain).

**Reviewer #1**:

- Are the objectives of the study clearly articulated with a clear testable hypothesis stated?
  Yes, the objectives of the study have been clearly articulated.

**Response:** We thank for this comment.

- Is the study design appropriate to address the stated objectives?
  There is a big problem with the study design. Indeed, all entomological studies were excluded which was a big mistake because all these diseases are transmitted by vectors meaning that measuring the impact of interventions should not be based only on serology but should be confirmed by the results of the published entomology studies

**Response:** We thank the reviewer for this important comment. We agree that entomological data provide critical insights into filarial transmission dynamics. While entomological studies were intentionally excluded from the systematic synthesis due to their fundamentally different outcomes compared to human prevalence estimates, we have now expanded the Discussion to include a narrative overview of key entomological evidence. This additional context highlights the persistence of competent vectors in endemic regions and supports the biological plausibility of the observed prevalence patterns, while maintaining the methodological coherence of the review.

- Is the population clearly described and appropriate for the hypothesis being tested? Is the sample size sufficient to ensure adequate power to address the hypothesis being tested?
  I do not think that the sample size is high enough to come to the conclusion described in this study. So, as recommended in question above, I have strongly recommended to the authors to include several other studies based on entomology only or having both a serology and an entomology component. There are plenty published studies having both components not cited here.

**Response**: We thank the reviewer for this thoughtful comment and agree that entomological data are essential for assessing transmission dynamics. Our study, however, was designed to examine how changes in diagnostic practices and research intensity influence reported human prevalence patterns, rather than to directly assess transmission interruption. To address the reviewer’s concern, we have expanded the Discussion to explicitly acknowledge the importance of vector infection data and added a dedicated limitation explaining why entomological studies were not integrated into the systematic synthesis. We also clarify that robust evaluation of concordance between human prevalence trends and vector infection rates would require temporally and geographically matched datasets, which are currently scarce and highly heterogeneous at the continental scale.

- Are there concerns about ethical or regulatory requirements being met?
  No, I do not think so.

**Response:** We thank the reviewer for confirming that ethical and regulatory requirements are appropriately met in this study.

- Regarding the results presented here, I can confirm that the analysis presented here matchs the analysis plan. The results are also really clear but for me these results are not presenting the real outcomes expected because plenty studies have been excluded.

**Response:** We thank the reviewer for highlighting this point. We have added a sentence to the Limitations section to clarify that only studies meeting pre-specified inclusion criteria—reporting raw prevalence data with confirmatory diagnostics—were included. We acknowledge that this approach led to the exclusion of numerous publications and may underrepresent some geographic regions or time periods. This clarification ensures that readers interpret the observed patterns in the context of study availability and methodological consistency.

- The concludions presented in this paper are supported by the data presenetd here. However, we have several limitations. A lot of published papers were not cited here.

**Response:** We acknowledge that not all published studies could be included in our review. Our search strategy focused on indexed literature available through PubMed, Scopus, African Index Medicus and ScienceDirect, and on studies providing quantitative human prevalence data. Studies published in local journals, in non-indexed formats, or in languages outside our search scope (e.g., French) were often inaccessible despite our best efforts. We have now explicitly noted this as a limitation in the manuscript (see Limitation section), emphasizing that the observed patterns may reflect both true epidemiological trends and structural inequalities in data availability.

- Entomology papers should be included as explained above and papers even containing both a serology and entomology components should be considered, which is not the case in the current evrsion of this manuscript.

**Response:** We agree that entomological data provide complementary information on transmission risk. However, the aim of this systematic review was to synthesize human prevalence data. Entomological studies were not included in the quantitative synthesis because they are highly heterogeneous in terms of methods, sampling periods, and geographic coverage, and integrating them with human prevalence data could introduce additional bias. Studies containing both serology and entomology components were assessed for inclusion, but only the human prevalence data were extracted for the systematic analysis. To ensure these data inform interpretation, we have discussed entomological findings in the Results and Discussion sections, highlighting their relevance to understanding vector presence, risk assessment, and limitations in linking human prevalence trends directly to interventions.

- Though this is an important paper showing progresses made so far regarding the impact of interventions for the four filarial diseases, there are several limitations to address. Without addressing these limitations it will be challenging to support the conclusion made by the authors of this manuscript.

Other comment

- Introduction: Burdens of onchocerciasis, Loa loa and Mansonellosis are missing. Please include tchem

**Response:** We thank the reviewer for this valuable suggestion. The Introduction has been revised to explicitly include quantitative estimates of the burden of onchocerciasis, loiasis, and mansonellosis. These figures highlight the substantial scale of these infections in Africa and reinforce the public health relevance of examining long-term prevalence trends in the context of diagnostic practices and uneven surveillance.

**Reviewer #2**

Major comments

1. Lack of Quantitative Synthesis: No pooled estimates, confidence intervals, or measures of heterogeneity (I²) are presented in the main text or figures, despite the use of ‘metaprop’.

**Response:** We thank the reviewer for pointing this out. Random-effects pooled prevalence estimates, 95% confidence intervals, and measures of heterogeneity (I², τ²) were indeed computed using the metaprop function for all included species and study periods. However, due to high heterogeneity across studies in terms of study design, diagnostic methods, and population characteristics, we chose to present only descriptive synthesis in the main text to avoid misinterpretation. All meta-analytic results, including pooled estimates, 95% confidence intervals, heterogeneity measures, and forest plots, are provided in the Appendix S4 (Table 1-2 S.4., Figures 1-4 S.4.) to allow full transparency. These supplementary materials provide the quantitative synthesis requested, without affecting readability of the main text.

2. Alternatively, authors should provide forest plots or summary tables of pooled prevalence estimates for key species/periods in supplementary materials (not as a raw data in S2).

**Response:** We thank the reviewer for the suggestion. We have added forest plots for each species and diagnostic method in the Appendix S4 (Fig. 1-4 S4) to visualise variability. Extreme heterogeneity (I² > 98%) precludes meaningful pooled estimates; thus, we maintain a descriptive approach

3. Formally test for subgroup differences (e.g., microscopy vs. molecular) using meta-regression, even if limited to more homogeneous subsets of studies. This is crucial to support claims that diagnostic method influences prevalence estimates.

**Response:** We thank the reviewer for this suggestion. We have performed random-effects meta-regression comparing microscopy and molecular diagnostic methods across all species with sufficient data (Appendix 4; Table 2 S4.). Results indicate that there are no statistically significant differences in pooled prevalence estimates between microscopy and molecular methods. We note that serological assays were not included in this comparison, as they measure exposure rather than active infection, and thus are not directly comparable to microscopy or molecular results. This limitation is now explicitly stated in the Methods and Results sections.

4. The figures 3-6 show prevalence trends but without measures of variance (e.g., 95% CIs) or accounting for differing numbers of studies per period. At least, error bar should be added.

**Response:** We have updated Figures 3–6 to include 95% confidence intervals for prevalence estimates for each time period.

5. The pie charts for diagnostic methods should be accompanied by absolute numbers (n) in the legend or on the charts themselves.

**Response:** We agree that including absolute numbers (n) would facilitate interpretation of the pie charts. However, to maintain figure readability and avoid visual overcrowding, we did not add counts directly on the charts. Instead, we provide the total number of studies included in each time period in the corresponding figure legends.

6. The maps included in figure 3-6 required distinctive colour contrast and also needed to include the keys to interpret the colours.

**Response:** The maps have been updated to improve colour contrast and now include clear colour keys to facilitate interpretation. In addition, the maps have been moved to separate figures to enhance overall readability.

7. The authors reported temporal trends, diagnostic methods, and geographic distribution of studies of species. However, geographical distribution should be a reflection of prevalence of the species in each region or country. This could be the ideal map for figures 3-6, instead of ‘geographical distribution of studies’. It is surprising that the authors did not analyze the prevalence of each species according to country or at least, representatives of top 5 or 10 countries with high prevalence should have been presented.

**Response:** We acknowledge the reviewer’s suggestion to present geographic patterns as prevalence estimates by country or region. However, our dataset covers a 25-year period and includes highly heterogeneous study designs, target populations, and diagnostic methods, which substantially limits the comparability of prevalence estimates across settings. Under these conditions, pooling prevalence by country for direct visual comparison on maps could be misleading. Therefore, we retained the maps to reflect the geographical distribution of included studies. Nevertheless, to address this point, we additionally calculated pooled prevalence estimates by country within defined time intervals and included these results in Supplementary Table S4 (Appendix 4; Table 3 S4).

8. The discussion lacks depth and centers on mere description. The discussion describes the problems (geographic bias, diagnostic limitations) but falls short of providing a novel or actionable synthesis.

**Response:** We thank the reviewer for this important suggestion. We have revised the Discussion to strengthen interpretation and highlight actionable insights. Specifically, we have added sentences emphasizing: 1/ the implications of geographic research gaps for undetected transmission and the need for targeted surveillance; 2/ the operational and resource-based determinants of diagnostic choices and their impact on observed prevalence trends; 3/ the interpretation of meta-analytic comparisons between microscopy and molecular diagnostics; 4/ the importance of leveraging existing health system structures, community engagement, and strategic use of diagnostics for sustainable elimination efforts; 5/ the need for operational research and surveillance for neglected pathogens such as *Mansonella* spp. These revisions are integrated throughout the Discussion, without altering the overall narrative flow. We believe these changes address the reviewer’s concern by providing a more nuanced and actionable synthesis of the findings.

9. No indication in the manuscript that the study protocol was registered in PROSPERO. This is not acceptable.

**Response:** We thank the reviewer for this comment. The systematic review was conducted according to a predefined protocol; however, the protocol was not registered in PROSPERO. All study selection criteria, data extraction methods, and analyses are fully described in the Methods section to ensure transparency and reproducibility of the review. We acknowledge that registration in PROSPERO is preferable and will be considered for future systematic reviews.

Minor Comments

10. No clear objective(s) of the study stated in both abstract & introduction.

**Response:** We thank the reviewer for highlighting the need for a clear statement of study objectives. We have now added explicit sentences in both the Abstract and Introduction to clearly define the aim of the study. Specifically, we state that the objective was to systematically review and synthesize population-based studies on human filariases in Africa between 2000 and 2025, examining temporal trends, diagnostic methods, and prevalence patterns across species and regions.

11. The keywords are redundant. Human filariasis; Neglected tropical diseases (NTDs); Africa; Lymphatic filariasis; Microscopy; Molecular diagnostics.

**Response**: We thank the reviewer for the suggestion. We have streamlined the keywords as recommended to improve clarity and avoid redundancy.

12. PECO Framework: The "Comparator" is vague. Specify that the comparison is implicit: trends over time and across diagnostic methodologies.

**Response:** We thank the reviewer for this comment. The comparator has been clarified to indicate that comparisons are implicit, examining trends over time and across diagnostic methods and regions.

13. Clarify what constitutes a "data point." Is it a unique pathogen-study-population combination? Ensure this is consistently applied.

**Response:** We thank the reviewer for this comment. We have clarified that a data point is defined as a unique pathogen-study-population-diagnostic method combination. This definition is now applied consistently throughout the manuscript.

14. The authors need to avoid assertive statements on MDAs, since the study did not compare or report MDAs coverage or status for each region.

**Response:** We thank the reviewer for this comment. We have revised the manuscript to avoid assertive statements regarding mass drug administration (MDA) impact. The text now describes observed prevalence patterns without implying causality, acknowledging that our review did not systematically collect MDA coverage or status for each region

15. Limitations: ‘Many regions of Africa, particularly Northern and parts of Southern Africa, are underrepresented in indexed databases due to limited research infrastructure, scarce funding, and reduced opportunities for local investigators to publish in high-impact journals.’ This is a vague statement without any evidence to support.

**Response:** We thank the reviewer for this comment. We have revised the statement in the Limitations section to reflect evidence on the uneven distribution of research output and publishing opportunities across Africa, supported by bibliometric studies. We emphasize that this limitation affects the geographic representativeness of available data without implying absence of disease in regions with fewer published studies.

**Reviewer #3**

The aim of the the systematic review is well articulated, below are some suggestions for clarifying concepts and improving the article:

1. Clarify what study designs qualify (e.g., community household surveys, school-based surveys, sentinel villages, cluster-based elimination surveys). Specify whether cluster-based LF/OV programme surveys are included and why.

**Response:** We thank the reviewer for this comment. We have clarified in the Methods that included studies were population-based surveys reporting human prevalence with confirmatory diagnostics, encompassing diverse settings such as school-based, household, and community surveys, as well as cluster-based evaluations of mass drug administration programs, provided that they reported original prevalence data.

2. Explain how multiple datapoints from the same study/site/timepoint were handled when producing any overall summaries or trends, and describe rules for selecting or aggregating overlapping estimates.

**Response:** We thank the reviewer for this important point. We have clarified in the Methods that multiple datapoints from the same study, site, or timepoint were extracted separately when they represented different age groups, diagnostic methods, or species. Overlapping prevalence estimates for the same population and timepoint were aggregated using the arithmetic mean to produce overall summaries, ensuring methodological variation is preserved while avoiding double-counting.

3. The inclusion criteria require confirmatory diagnostics, yet exposure-based serology (e.g., Ov16 IgG4, Pan-IgG-SXP-1) is included. Define which tests were treated as evidence of active infection vs exposure, whether antigen tests were handled differently from antibody tests, and how heterogeneous endpoints were standardised. At minimum, categorise endpoints explicitly (mf microscopy, antigen detection, antibody/serology, molecular) and pre-specify how each contributes to analyses.

**Response:** We thank the reviewer for raising this important point. Our systematic review is designed to provide a long-term, continent-wide perspective on trends in human filarial prevalence and research activity, rather than to generate precise current prevalence estimates. For this reason, we have combined all eligible data points across diagnostic methods. While microscopy and molecular assays indicate active infection, serological tests reflect exposure; including all data allows us to capture temporal and methodological patterns that would be missed if focusing solely on one type of diagnostic. To improve transparency, we now clearly indicate the dominant diagnostic method for each period using pie charts overlaid on prevalence points and provide detailed breakdowns in the supplementary tables. This approach ensures that readers can interpret observed prevalence trends in the context of changing diagnostic practices and research intensity

4. If metaprop was used, describe the meta-analytic model (e.g., random-effects), weighting, transformations, and heterogeneity outputs. If not, remove the pooled-estimate claim and describe a purely descriptive approach. Also specify whether 5-year means were sample-size-weighted or unweighted and justify the choice.

**Response:** We thank the reviewer for this comment. While we did compute formal pooled prevalence estimates using the metaprop function in the meta package (including random-effects modeling, 95% confidence intervals, and measures of heterogeneity such as I² and τ²), these analyses are presented in the Appendix 4 (Table 1-2 S4, Figures 1-4 S4) due to the high heterogeneity of included studies in terms of design, diagnostic methods, population age groups, and sampling periods. In the main text, we focus on a descriptive synthesis, calculating pooled prevalence values for five-year intervals to provide a long-term overview of temporal trends. This approach allows visualization of changes in reported prevalence while avoiding inappropriate pooling of fundamentally incomparable data points. Maps and overlaid pie charts illustrate the proportion of studies using each diagnostic method, giving readers context for interpreting observed trends.

5. If linking prevalence declines to MDA, extract and incorporate programme exposure variables (years/rounds/coverage/baseline endemicity/program phase) or transparently classify settings as MDA-implemented vs no/unknown using study context and/or WHO/ESPEN data. Otherwise, soften causal language in Methods-driven interpretation.

**Response:** We thank the reviewer for highlighting the need for clarity regarding MDA exposure. Detailed programmatic data (rounds, coverage, baseline endemicity) were not systematically extracted for this review. Consequently, we have softened causal language in the manuscript, describing temporal declines as coinciding with MDA implementation rather than attributing causality.

6. The results clearly presents the analysis plan, the limitations are clearly articluated. One limitation was dart of publications. Below are some suggestions for improvement:
   6.Combining the mf prevalence, antigen prevalence, and antibody prevalence into the prevalence trend makes the outcome ambiguous. Present separate trend results by diagnostic endpoint (or stratified lines/sensitivity analyses) and interpret any combined line only as a heterogeneous average.

**Response:** We appreciate this observation. Our systematic review is designed to provide a long-term, continent-wide perspective on trends in human filarial prevalence and research activity, rather than precise current prevalence estimates. To capture temporal and methodological patterns, we combined all eligible data points across diagnostic methods, acknowledging that microscopy and molecular assays indicate active infection, while serology reflects exposure. To enhance transparency, we now indicate the dominant diagnostic method for each period using pie charts overlaid on prevalence points and provide detailed breakdowns in Supplementary Tables. In the revised manuscript, we clearly state that combined prevalence lines should be interpreted as a heterogeneous average rather than a precise measure of current infection burden.

7. Figures currently show point estimates only; add confidence intervals/uncertainty bands (or IQRs/bootstrapped intervals if descriptive) and report the number of studies and total sample size contributing to each 5-year interval, especially important for sparse pathogens like *L. loa* and *Mansonella*.

**Response:** We thank the reviewer for this important suggestion. In response, the main figures (Figures 3–6) have been updated to include 95% confidence intervals for each 5-year mean prevalence, as well as the number of contributing studies and total sample size per interval. Pie charts continue to show the distribution of diagnostic methods. This ensures transparency regarding data variability and sparsity, particularly for less frequently studied pathogens such as *L. loa* and *Mansonella* spp., while maintaining figure clarity.

8. If metaprop outputs were generated, present them (at least in supplementary forest plots by species × endpoint × interval) and report heterogeneity statistics; if not, remove references to pooled prevalence estimates.

**Response:** We thank the reviewer for this comment. While included studies remain highly heterogeneous in terms of design, population age groups, diagnostic methods, and sampling periods, we have now generated meta-analytic outputs using metaprop. Pooled prevalence estimates, 95% confidence intervals, and heterogeneity statistics (I², τ²) are presented in Supplementary Tables S1–S4 and forest plots by species × endpoint (Appendix 4 Figures 1-4 S4). In the main text, we retain descriptive statistics with mean prevalence values for 5-year intervals to provide a clear overview of temporal trends. Maps and pie charts continue to illustrate the distribution of diagnostic methods, aiding interpretation of observed patterns while avoiding overcrowding the figures.

9. Based on the extracted data, the results suggest there were “declines observed during the MDA era,” but they do not directly attribute these declines to MDA without specific programme exposure information. The wording should be adjusted to say “consistent with” or “occurring alongside.”

**Response:** We agree that causal attribution is not possible given the lack of detailed programme exposure data for all sites. Accordingly, we have softened the language in the Discussion

10. The conclusion supports data presented, except for the information on MDA which wa not part of the data extracted and analysed. The limitation is well articluated and the article is of public health relevance partiulary within the NTD community, highligtging the gap in survellience and diagnostics.

**Response:** We thank the reviewer for highlighting this point. We agree that the systematic review did not extract or analyse detailed programme-level MDA variables (e.g., rounds, coverage, baseline endemicity). In response, we have softened the language throughout the Discussion section to reflect this, changing statements that previously implied causality to phrasing such as “consistent with MDA implementation” or “occurring alongside MDA programs.” This ensures that conclusions remain fully supported by the data analysed while acknowledging broader public health interventions and their temporal overlap with observed trends.

11. This article is significant particularly for its focus on practical relevance and highlighting how limited surveillance and diagnostics can hinder elimination efforts and lead to incorrect interpretations of prevalence trends. It is notable for covering a broad 25-year period and analysing multiple pathogens, such as LF, onchocerciasis, loa-loa, and mansonellosis, especially considering their co-endemicity and potential diagnostic cross-reactivity.

**Response:** We thank for their positive feedback and are glad that the practical relevance, multi-pathogen perspective, and consideration of diagnostic challenges in our 25-year analysis are appreciated.

**Reviewer #4**

This paper describes the changes in the epidemiology of filariases in Africa from 2020 to 2025. Overall the paper is well written and clearly described. Below are some comments to improve the content and relevance of the paper.

-When describing the human filariases, it is perhaps recommended to say loiasis and mansonellosis, in line with lymphatic filariasis and onchocerciasis. This should be addressed in the abstracts and throughout the text. If mentioning the scientific names of the parasites, then all should be mentioned. Not the disease in some cases and the parasites in others.

- Lines 69 - 72. It might be useful to indicate the different Mansonella species known to cause human disease in Africa.

- Line 99: *L. loa*

- Figure 1: Check the spelling of "papers" and "sufficient".

- Line 175: Please provide the exact number of participants. Also, it is not clear what the data points refer to. Please clarify.

- Figure 2: A colored image would be helpful. Kindly reverse the colors for the countries with little data to have the lighter colors and those with more data to have the deeper colors.

- Figures 3 - 6: A legend should be provided for the maps. Same comments on the colors also apply here.

- Result on *Mansonella* spp.: Could these be further elaborated to indicate the species that are most common?

- Line 254: Check the spelling of "Discussion"

- Results: it would have been useful to present a country by country analysis of each disease and the decline in prevalence. This would be important for disease control programmes.

- It would be very interesting and useful to know how the results compare to the ESPEN data.

**Response:** We thank for their constructive and encouraging feedback, and we have addressed the suggested clarifications, spelling corrections, figure improvements, and additional details on *Mansonella* species and data presentation throughout the manuscript. We have added Table 3 S4, which presents the observed prevalence for each parasite by country and time period, alongside the ESPEN-reported endemicity status and MDA implementation. This allows a direct comparison with ESPEN data and highlights potential discrepancies between reported MDA coverage and observed prevalence, which are discussed in the manuscript
